# INAAC: An affinity chromatography strategy enabling characterization and quantification of influenza neuraminidase antigens in vaccines

**DOI:** 10.1016/j.jbc.2026.113138

**Published:** 2026-05-12

**Authors:** Hyeog Kang, Anna M. Borowska, Tapan Kanai, Jin Gao, Hai Yu, Xi Chen, Jason Gorman, Dirk-Jan Slotboom, Robert Daniels

**Affiliations:** 1 Division of Viral Products, Center for Biologics Evaluation and Research, Food and Drug Administration, Silver Spring, Maryland, USA; 2 Faculty of Science and Engineering, Groningen Biomolecular Sciences and Biotechnology, Membrane Enzymology Group, University of Groningen, Groningen, the Netherlands; 3 Department of Chemistry, University of California, Davis, California, USA

**Keywords:** enveloped virus surface antigens, influenza virus vaccines, influenza surface antigens, influenza NA active-site affinity chromatography, INAAC, NA purification, structure of native NA vaccine antigens, zanamivir, NA vaccine standards

## Abstract

Seasonal influenza vaccines are commonly produced using viruses containing hemagglutinin (HA) and neuraminidase (NA) antigens from recommended strains. However, NA amounts are not monitored due to lack of strategies for isolating NA reference antigens from viruses, limiting the ability to evaluate any contributions from NA for more than 50 years. Here, we developed an influenza neuraminidase active-site affinity chromatography (INAAC) strategy that uses an active-site binding antibody to isolate functional NAs from influenza A and B vaccine strains. INAAC recovered 20 to 60% of detergent-solubilized NA activity from vaccine viruses and recombinant sources, with CaCl_2_ elution proving most effective. ELISA results with the isolated NA from the H1N1 strain and recombinant full-length N1 showed that a commercial vaccine contains functional N1 and H1 at a ratio of ∼1:10. Structure determination of the isolated N1 by cryo-electron microscopy confirmed the native tetrameric conformation and provided insight into the stalk conformation of a virus-derived NA. Finally, comparative analyses of NAs isolated from recent egg-propagated vaccine strains (H1N1, H3N2, and type B) revealed a unique proteolytic susceptibility of type B NA and strain-specific differences in sialic acid affinities and catalytic rates. These results demonstrate that INAAC supports multiple applications from NA structural analysis to producing NA vaccine antigens and reference standards, addressing longstanding challenges for incorporating NA into influenza vaccine development and quality control.

Proteins on the surface of envelope viruses are often essential for replication, making them attractive targets for therapeutics and vaccines. The surface of influenza viruses contains two key glycoproteins, hemagglutinin (HA) and neuraminidase (NA) (1, 2). HA facilitates viral entry by binding sialic acid-conjugated cell surface receptors (3, 4, 5), while NA contributes to viral movement by cleaving local sialic acid residues to prevent persistent HA binding (6, 7, 8, 9). Currently, all licensed influenza vaccines primarily target the receptor-binding function of HA (10). However, numerous studies have shown that NA activity inhibitory (NAI) antibodies are also protective (11, 12, 13, 14), suggesting the suboptimal efficacy of influenza vaccines could be improved by incorporating NA antigens.

The historical bias toward HA in influenza is partly related to its relative abundance and role in initiating infection and partly related to historical methodological limitations. The first licensed influenza vaccines simplified antigen production and isolation by using inactivated viruses that contain high amounts of HA (15, 16). The development of these vaccines coincided with George Hirst’s invention of the hemagglutination inhibition (HI) assay, which showed vaccines elicit antibodies against the receptor-binding function of HA in a dose-dependent manner (17, 18). Consequently, HI became, and remains, the standard assay for measuring influenza vaccine responses (19). Methods for ensuring consistent HA content, such as Single Radial Immunodiffusion, were implemented much later (1978), as techniques for isolating HA from viruses were not available until 1972 (20).

Introducing new or additional antigens to vaccines targeting enveloped viruses remains challenging because simple processes for isolating surface (membrane) proteins from these viruses are lacking. In addition, some surface proteins like influenza HA and NA are also constantly evolving (21, 22, 23, 24), making the development of isolation strategies even more chalenging. The hypothesis that influenza vaccine efficacy can be improved by supplementing vaccines with exogenous NA antigen exemplifies these issues. Although first proposed by the Kilbourne lab in the 1990s (25), this hypothesis has yet to be rigorously tested due to slow progress in developing methods for producing stable NA antigens (26, 27, 28, 29), measuring NAI antibodies (30, 31, 32, 33, 34, 35) and quantifying NA content in vaccines (36, 37, 38).

NA is a labile Ca^2+^-dependent sialidase that functions as a tetramer (39, 40), further complicating analysis and isolation. In addition, NA content in viruses is much lower than HA (41, 42). NA assembles into tetramers by a cooperative process involving the *N-*terminal transmembrane (TM) domain, stalk and the enzymatic head of each protomer (43, 44, 45, 46). However, the first structure of NA was determined with enzymatic head domains isolated from virus particles by proteolytic cleavage (47, 48). These isolated heads, lacking the TM and all but ∼5 residues of the stalk, reportedly retained ∼66% of the initial activity when assayed using the glycoprotein substrate fetuin, suggesting the TM and stalk regions are dispensable for catalytic activity. This historical precedent may have inadvertently discouraged the development of methods to isolate and study full-length NA, as structural information for the full-length protein is still lacking nearly 50 years later.

Today, NAs are often produced as recombinant secreted chimeras isolated by *N-*terminally fused affinity tags or multi-step chromatography (28, 49, 50, 51). Recently, multiple active-site-binding ligands have been identified or synthesized that can bind a broad spectrum of NAs (12, 13, 14, 52), which are ideal for creating affinity approaches to purify unmodified NAs. In this study, we used an immobilized monoclonal antibody (MAb) ligand to develop an Influenza Neuraminidase Active-site Affinity Chromatography (INAAC) strategy. We used INAAC to isolate and characterize native full-length NA antigens from recent vaccine strains (H1N1, H3N2, and type B) that were produced in insect cells or using viruses propagated in eggs. Our results show that INAAC successfully yields enzymatically active full-length NAs and reveal that NA from the type B virus is uniquely susceptible to proteolysis. Analysis by cryo-electron microscopy (cryo-EM) and ELISA indicates that the isolated viral NAs are in their native tetrameric conformation and are suitable to quantify functional NA in commercial vaccines. These results demonstrate the utility of this simple procedure for multiple applications, from manufacturing NA antigens and standards to obtaining structural information for native NAs isolated from viruses.

## Results

### Development of an influenza NA active-site affinity chromatography (INAAC) strategy

**Figure 1:**
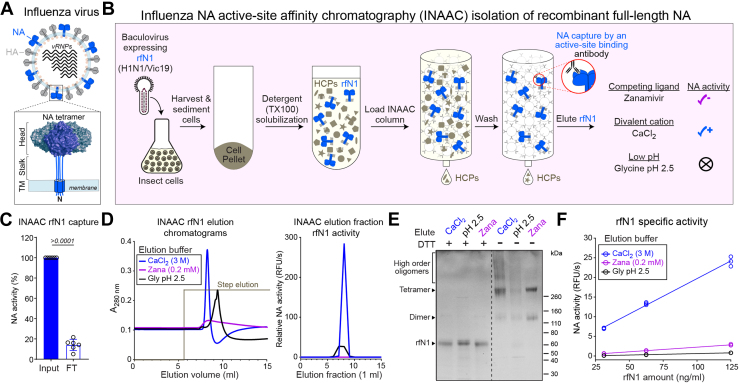
**Characterization of rfN1 isolated with different INAAC strategies.***A*, Influenza virus diagram displaying the location of the HA and NA surface antigens. Inset shows NA tetramer topology in the viral membrane. Regions corresponding to the NA enzymatic head, stalk and transmembrane (TM) domains are indicated. *B*, Experimental scheme for isolating and eluting rfN1 from insect cells by INAAC. rfN1 was captured by an antibody that binds the conserved active site, enabling host cell protein (HCP) removal. Impact of the elution approaches on rfN1 activity are summarized. *C*, rfN1 capture was monitored by NA activity using equal volumes of solubilized insect cell lysate (Input) and INAAC column flow throough (FT). FT activity was normalized to input activity that was set to 100%. Bars show means ± standard deviation (SD) from three independent experiments run in duplicate. *p* value is from an unpaired student *t* test. *D*, Representative chromatograms (*left panel*) of rfN1 eluted by the indicated approach. NA activities (*right panel*) are shown from equal volumes of the 1 ml elution fractions. *E*, Coomassie stained SDS-PAGE gel of rfN1 eluted with CaCl_2_, low pH or zanamivir (Zana). Equal rfN1 amounts (∼2 μg) were treated or untreated with dithiothreitol (DTT) prior to resolution. Bands corresponding to rfN1 monomers, dimers, SDS resistant tetramers and higher order oligomers are indicated. *F*, Specific activities of rfN1 eluted by CaCl_2_, low pH, or Zana, were measured in triplicate using MUNANA. All values are displayed.

Enveloped virus surface antigens like influenza HA and NA are constantly evolving membrane proteins that are challenging to isolate (Fig. 1*A*). To address these issues for influenza NA, we focused on leveraging active-site binding ligands to develop a procedure capable of isolating NAs from a broad range of influenza A and B viruses. As a proof-of-concept, we created a column containing Sepharose beads cross-linked to a recently identified MAb (FNI-9) that binds to the NA active site through substrate mimicry (12). We then used the column to capture detergent-solubilized recombinant full-length NA (rfN1) from the H1N1 vaccine strain A/Victoria/2570/2019 that was expressed in insect cells and tested multiple approaches for eluting the NA in a functional state (Fig. 1*B*). These included the NA inhibitor zanamivir (Zana) because it likely functions as a competing ligand due to its high affinity with an ∼5 nM half-maximal inhibitory concentration for NAs (41, 53), CaCl_2_ because divalent cations can weaken electrostatic protein-antibody interactions (54) and Ca^2+^ stabilizes NA (40), and a pH 2.5 glycine buffer control which disrupts antibody and NA structures (55).

After passing the Triton-X 100 (TX100)-solubilized rfN1 through the INAAC column, ∼15% of the input activity was found in the flow-through (Fig. 1*C*), indicating ∼85% rfN1 capture. During the elution comparisons, we observed a broad diffuse peak with Zana, a sharp peak with CaCl_2_, and an intermediate peak with the pH 2.5 buffer that included a small early shoulder (Fig. 1*D*, left panel). The CaCl_2_ peak fraction contained high NA activity, whereas the low pH fractions showed lower activity that was limited to the early elution shoulder (Fig. 1*D* right panel). The Zana elution fractions contained no activity, as expected.

We combined the peak fractions based on the absorbance at 280 nm, exchanged the buffer by dialysis, and resolved equal protein amounts by SDS-PAGE. The reduced (RD) rfN1 samples from all three eluates showed similar band profiles and purity (Fig. 1*E*Table S1). Under nonreducing (NR) conditions, CaCl_2_ and Zana-eluted rfN1 resolved mainly as intermolecular disulfide-bonded dimers formed by Cys49 in the stalk (45), and SDS-resistant tetramers. In contrast, the low pH-eluted rfN1 bands were less intense, indicative of protein loss due to aggregation. Most importantly, rfN1 eluted with CaCl_2_ displayed 10-fold higher specific activity than that eluted with Zana and 100-fold higher than that with the low pH elution (Fig. 1*F*), indicating CaCl_2_ is the most effective strategy for recovering functional NA.

### Characterization of full-length NA isolated from an H1N1 virus by INAAC

**Figure 2:**
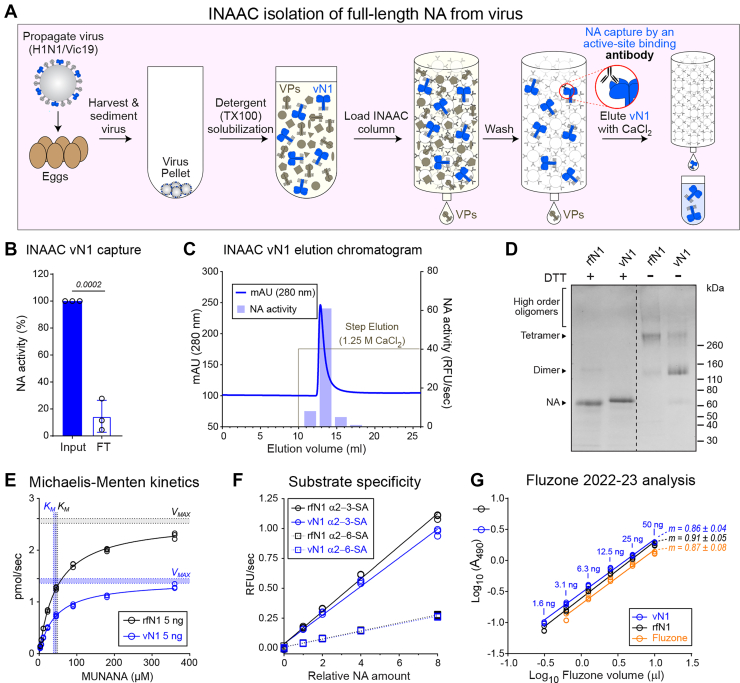
**Characterization of NA from an H1N1 vaccine virus isolated by INAAC.***A*, INAAC strategy for isolating full-length N1 (vN1) from an egg propagated H1N1 vaccine virus. Following vN1 capture, viral proteins (VPs) were removed by washing and vN1 was eluted with CaCl_2_. *B*, vN1 capture was monitored by measuring NA activity in equal volumes of detergent solubilized virus (Input) and column FT. Each FT activity was normalized to the input activity that was set to 100%. Bars show means ± SD from three independent experiments. *p* value is from an unpaired student *t* test. *C*, Representative chromatogram of vN1 eluted using CaCl_2_. Bars show relative NA activity in the collected fractions. *D*, Coomassie-stained SDS-PAGE gel of INAAC isolated rfN1 and vN1 eluted with CaCl_2_. Equal protein amounts (∼2 μg) were treated with or without DTT prior to resolution. Bands corresponding to monomers (NA), dimers, SDS resistant tetramers and higher order oligomers are indicated. *E*, Michaelis-Menten kinetic analysis of equal amounts of CaCl_2_ eluted rfN1 and vN1. Assays were run in triplicate with indicated MUNANA concentrations and all data points are shown. *Dotted lines* correspond to the 95% confidence interval (CI) of the calculated Michaelis Constant (*K*_*M*_) and maximum velocity (*V*_*Max*_). *F*, Sialyl linkage-specific catalytic activity of rfN1 and vN1 was measured using α2–3- and α2–6-linked sialosides. Protein amounts were standardized based on α2–3-sialyl cleaving activity and serially diluted two-fold. Data from three independent runs are shown. *G*, N1 sandwich ELISA results showing the log-log linearity of a serially diluted expired Fluzone lot from the 2022-23 season and the indicated amounts of rfN1 and vN1. Data from an assay run in triplicate are shown with the linear regression slope (*m*) ± 95% CI.

We applied a similar INAAC strategy to isolate NA directly from the H1N1 vaccine strain A/Victoria/2570/2019. The approach involved concentrating egg-propagated viruses by sedimentation, solubilizing the viral NA (vN1) with TX100, followed by INAAC capture and elution with 1.25 M CaCl_2_ (Fig. 2*A*). We reduced the CaCl_2_ concentration from 3 M based on the observation that rfN1 eluted in previous trials at ∼35% of the peak conductance (*e.g.*, ∼1 M). Similar to rfN1, vN1 activity in the INAAC flow through was ∼20% of the input, suggesting ∼80% was captured (Fig. 2*B*). We then step eluted vN1 with 1.25 M CaCl_2_, combined the peak NA activity fractions (Fig. 2*C*), and exchanged the buffer.

On Coomassie-stained SDS-PAGE gels, the isolated vN1 under RD conditions showed a similar purity (∼80%) to rfN1 but a slower mobility (Fig. 2*D* and Table S1). The mobility difference is likely due to the attachment of *N-*linked glycans to the NA on virus propagated in eggs (vN1) that are larger than those attached to rfNA expressed in insect cells (27, 56, 57). Unexpectedly, vN1 resolved under NR conditions as monomers, dimers and SDS-resistant tetramers, whereas rfN1 mainly resolved as dimers and SDS-resistant tetramers, suggesting some vN1 is not properly assembled. Supporting this observation, a kinetic analysis at pH 7 with the synthetic substrate 2-(4-methylumberllifery)-α-D-*N*-acetylneuraminic acid (MUNANA) in a buffer containing 1 mM CaCl_2_ showed that the apparent catalytic rate (*i*.*e*. *V*_*max*_) of vN1 is ∼50% lower even though the substrate affinities (*i*.*e*. Michaelis constants (*K*_*m*_)) are similar (Fig. 2*E*). When adjusted for activity, both proteins showed similar specificities for cleaving α2−3- and α2−6-linked sialic acids (Fig. 2*F*). Together, these results indicate that either more vN1 was deactivated during the INAAC procedure, vN1 has a lower catalytic rate, or viruses contain a large fraction of improperly assembled NA.

### Measuring vaccine NA content with INAAC-isolated NA

We tested if INAAC-isolated vN1 could serve as a reference antigen or standard for measuring NA content in commercial vaccines by using a stability-indicating N1 sandwich ELISA (Fig. S1) to analyze an expired Fluzone lot from the 2022-23 season alongside known vN1 and rfN1 amounts (Fig. 2*G*). Results from the vN1 and rfN1 titrations were very similar (Figs. S1*B* and S2*A*), showing log-log linearity across a protein range of 1.6 to 50 ng (Table S2) and parallelism (*e.g*. similar slopes) to vaccine dilutions ranging from 0.625 to 10 μl (Fig. 2*G* and S2*B*). Using the vN1 standard curve, we estimated that a 0.5 ml dose of the 2022-23 expired Fluzone lot contains ∼ 1.4 μg of N1 (Table S3), resulting in an N1:H1 ratio of ∼1:10, consistent with previous estimates for viruses (42, 58).

### Structural analysis of INAAC-isolated vN1

**Figure 3:**
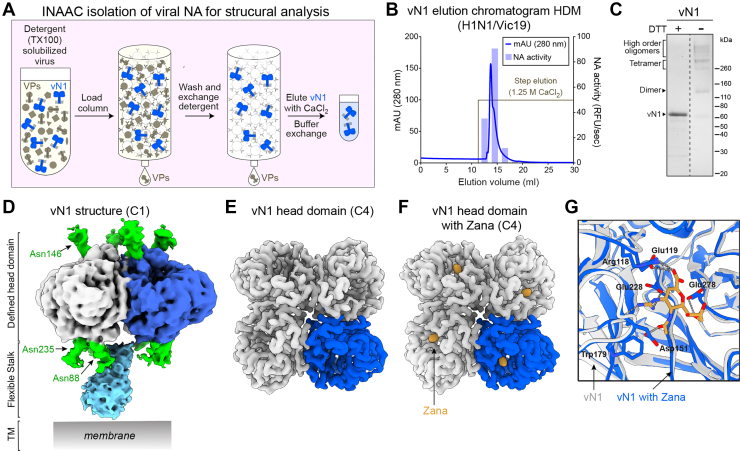
**Cryo-EM structural analysis of NA isolated from an H1N1 vaccine virus.***A*, Diagram illustrating the detergent exchange introduced into the INAAC strategy to enable vN1 analysis by cryo-EM. *B*, Chromatogram of the detergent exchanged vN1 eluted using CaCl_2_. Bars show relative NA activity profiles in the collected fractions. *C*, Coomassie-stained SDS-PAGE gel of the isolated vN1 used for cryo-EM structural analysis. vN1 (∼2 μg) was treated with or without DTT prior to resolution. Bands corresponding to monomers (NA), dimers, SDS resistant tetramers and higher order oligomers are indicated. *D*, Cryo-EM density map of vN1 reconstructed with C1 symmetry, displayed at low contour threshold (σ2) and viewed from the side. The map is colored *gray*, with a single head domain protomer in *blue*, stalk region in *light blue*, and densities of the conserved head domain *N*-linked glycans in *green*. Predicted location of the transmembrane region is indicated by a gray gradient. *E* and *F*, Cryo-EM density maps of tetrameric vN1 head domains reconstructed with C4 symmetry in the absence (*E*) and presence (*F*) of Zana, viewed from the top of the tetramer. A single protomer is in *blue* and the other subunits in *gray*. Bound Zana density is depicted in *yellow*. *G*, Superposition of the vN1 head domain protomer in the absence and presence of Zana. The apo vN1 structure is *gray*, Zana-bound vN1 is *blue*, and the bound Zana is depicted in *yellow*. Key residues that interact with Zana are highlighted, including Asp151 in the 150-loop.

To examine the structure of the isolated vN1, we used single-particle cryo-EM. Initial images showed high background, which was reduced by replacing TX100 with *n*-hexadecyl-β-*D*-maltoside (HDM) in the wash and elution buffers (Fig. 3*A*). Following this change, vN1 still eluted in a clear peak with activity (Fig. 3*B*), showed good purity on RD SDS-PAGE gels (Fig. 3*C*), and the expected oligomeric states under NR conditions. On the cryo-EM micrographs, vN1 particles were readily visible, enabling alignment into 2D classes (Fig. S3). The resulting 2D class averages showed a well-defined enzymatic head domain with substantially weaker signal for the stalk and even less signal for the transmembrane domain, indicating these regions are flexible relative to the head.

The intial cryo-EM map for the isolated vN1 was obtained without imposing symmetry. It showed that vN1 maintains its native tetrameric conformation with a well-resolved head domain that sits on a flexible stalk region with weaker, more diffuse density indicative of significant motion relative to the head (Figs. 3*D* and S3, and Table S4). vN1 encodes 3 *N*-linked glycan sites in the head domain (N88, N146 and N235) that are highly conserved in H1N1 viruses (59, 60), and five in the stalk (N42, N50, N58, N63 and N68) that are common in H1N1 2009 pandemic viruses (61). Our reconstructed map showed protruding density near the Asn (N) residue of the three conserved head domain sites, confirming they are glycosylated during viral replication in eggs (Fig. 3*D*).

In the C1 reconstruction, the stalk region appeared as unresolved, featureless density (Fig. 3*D*). The reduced resolution is likely due to the stalk’s intrinsic flexibility, which was apparent in an analysis of the C1 conformational variability (Movie S1), and the disordered nature of the ∼20 *N-*linked glycans. Despite the low resolution, the observed density suggests that the stalk attaches to the head by a flexible, narrow linker and does not adhere to C4 symmetry as it is tilted relative to the fourfold symmetry in the head. To obtain a final map, we imposed C4 symmetry and built a head domain model with 3.58 Å resolution (Figs. 3*E* and S3, and Table S4)

### Impact of zanamivir binding on the vN1 structure

NAs that are less stable in high CaCl_2_ concentrations than vN1 can potentially be eluted with Zana for structural analysis. Therefore, we determined a 3.48 Å resolution cryo-EM structure of vN1 in complex with Zana by focusing the reconstruction on the head domain and imposing C4 symmetry (Figs. 3*F* and S4, and Table S4). In each vN1 protomer active site, we observed distinct densities corresponding to bound Zana. Superposition of the vN1 structures with and without Zana showed high similarity (global C-α RMSD of 0.626 Å), supporting that CaCl_2_ or Zana can be used for NA elution depending on downstream applications.

NAs are classified into two functional groups based on the 150-loop conformation: open (group 1) or closed (group 2). However, closed states are commonly observed upon inhibitor binding (62, 63). While N1 is generally in group 1, an overlay of the two vN1 structures showed that the 150-loop is in a closed state whether Zana is absent or present (Fig. 3*G*). A closed 150-loop without Zana was also previously observed for a highly similar recombinant NA (global C-α RMSD of 1.4 Å) from the H1N1 strain A/California/04/09 (50), suggesting a closed 150-loop is a general property of H1N1 pandemic strain NAs. However, we did note lower density for the 150-loop in the absence of Zana, indicating it likely is flexible and that Zana binding may stabilize a preexisting active site conformation.

### Modifying INAAC for recent H3N2 and type B vaccine strains

**Figure 4:**
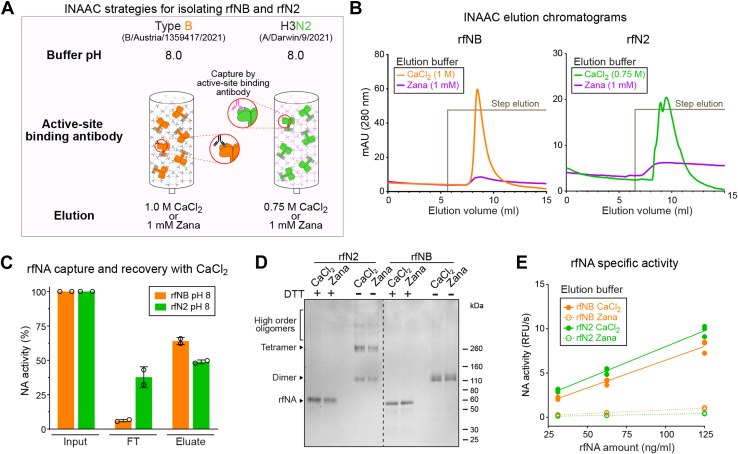
**INAAC modifications for isolating rfNAs from recent H3N2 and type B vaccine viruses.***A*, Diagram showing the different buffer pH, antibody ligands, CaCl_2_ and Zana concentrations used for INAAC isolation of NAs from recent H3N2 and type B vaccine viruses. *B*, Chromatograms of the rfNB (*left*) and rfN2 (*right*) elution profiles with the indicated CaCl_2_ or Zana concentrations are displayed. *C*, Capture and recovery of each rfNA were determined by measuring total NA activity in the INAAC input, FT and eluate. Activity values were normalized to the input that was set to 100%. Bars show means ± SD from two independent experiments. *D*, Coomassie-stained SDS-PAGE gel of the isolated rfNB and rfN2. Equal protein amounts (∼2 μg) were treated with or without DTT prior to resolution. Bands corresponding to monomers (rfNA), dimers, tetramers and higher order oligomers are indicated. *E*, Specific activities of rfNB and rfN2 eluted by CaCl_2_ or Zana were measured in triplicate using MUNANA. All values are displayed.

To encompass all currently recommended influenza virus vaccine strains (H1N1, H3N2 and B Victoria), we optimized INAAC using insect cell-expressed recombinant full-length NAs (rfNAs) from the A/Darwin/9/2021 (H3N2) and B/Austria/1359417/2021 vaccine strains. Initially, we performed several pilot experiments that took into consideration previous results showing that the N2 affinity for the substrate reporter MUNANA increased over the physiological pH range of 6 to 8 (55), a point mutant (R27D) in the capture MAb can increase the affinity for N2 (30), and the possibility of high CaCl_2_ ionic strength disrupting the NA structure over time. These initial tests confirmed rfN2 capture efficiency was improved with the R27D MAb as well as with a pH 8 buffer, and the latter also supported high rfNB capture with the unmodified MAb. Additional gradient elution tests indicated 0.75 M CaCl_2_ was sufficient for rfN2 elution and 1 M CaCl_2_ for rfNB elution, whereas prolonged CaCl_2_ exposure experiments (22 h at room temperature) showed that the three rfNAs remain relatively stable between 20 to 375 mM CaCl_2_ with pH-dependent drops at higher concentrations, further supporting the use of pH 8 for rfNB and rfN2 (Fig. S5). Based on these results, we modified the INAAC isolation procedure for rfN2 and rfNB by applying a pH 8 buffer (Fig. 4*A*), using the R27D MAb for rfN2, and pre-adding two fraction volumes of buffer in the collection tubes to rapidly dilute CaCl_2_ to below 375 mM. We also increased the Zana concentration five-fold to 1 mM to try to improve the elution efficiency and used a detergent *n*-tetradecyl-β-*D*-maltoside (TDM) with a shorter aglycon than HDM and increased solubility at cooler temperatures.

Profiles of the TX100-solubilized rfNB and rfN2 eluted in CaC1_2_ and Zana with TDM (Fig. 4*B*) were similar to rfN1. CaCl_2_ elution produced a strong peak for rfNB and a broader double peak for rfN2, indicative of more than one population, whereas Zana resulted in broad peaks. With CaC1_2,_ we recovered ∼65% of the total rfNB activity and ∼50% of the rfN2 activity, which were consistent with the capture efficiencies (Fig. 4*C*). RD and NR SDS-PAGE profiles of rfN2 and rfNB eluted with CaCl_2_ or Zana were indistinguishable (Fig. 4*D*), but both Zana-eluted proteins still displayed 10 to 20-fold lower specific activity despite extensive dialysis (Fig. 4*E*), supporting that CaCl_2_ is more effective for recovering enzymatically active NAs.

### INAAC validation with seasonal influenza vaccine strains

**Figure 5:**
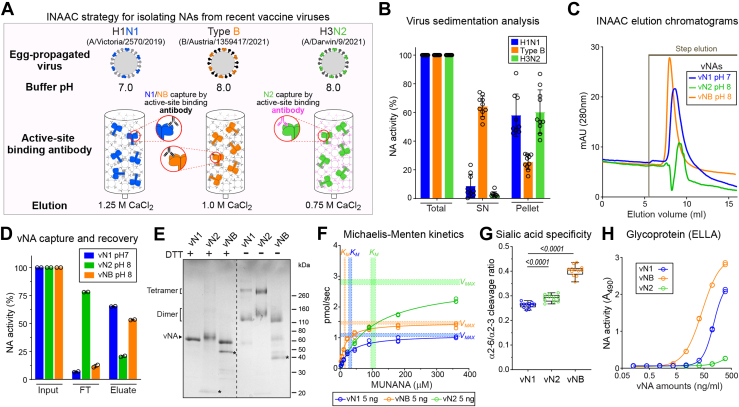
**Characterization of INAAC isolated vNAs from H1N1, H3N2 and type B vaccine viruses.***A*, INAAC strategies for isolating vNAs from the indicated vaccine viruses recommended for the 2022-23 season. *B*, NA activity from the indicated viruses was measured in the allantoic fluid of 10 eggs per strain and in the supernatant (SN) and detergent solubilized pellet after sedimentation. For each egg, total NA activity in the allantoic fluid was set to 100%. Bars show means ± SD with all the individual data points. *C*, Chromatograms of the vNA elution profiles with CaCl_2_. *D*, Capture and recovery of each vNA was determined by measuring total NA activity in the INAAC input, FT and eluate. Values were normalized to the input activity that was set to 100%. Bars show means ± SD from one experiment analyzed in duplicate. *E*, Coomassie-stained SDS-PAGE gel of the isolated vNAs. Equal protein amounts (∼2 μg) were treated with or without DTT prior to resolution. Bands corresponding to vNA monomers (vNA), dimers and tetramers are indicated. Asterisks indicate lower molecular weight bands likely corresponding to vNB proteolytically cleaved in the stalk. *F*, Michaelis-Menten kinetic analysis of equal amounts of each vNA with MUNANA run in triplicate. All data points are displayed. *Dashed lines* correspond to the 95% CI of the calculated *K*_*M*_ and *V*_*Max*_. *G*, Sialyl linkage-specific cleavage activity of the vNAs were measured with an enzyme coupled assay using α2–3- and α2–6-linked sialosides (Fig. S7) and the α2–6 to α2–3 cleavage ratios are displayed for each vNA. Data are from ratios of all dilutions within the linear range of the assay from three independent runs. *p* Values are from an unpaired student *t* test. *H*, Specific activities of the vNAs on bovine fetuin were measured by an enzyme-linked lectin assay (ELLA). Representative data from an experiment run in duplicate are shown.

For a final validation, we propagated H3N2, type B, and H1N1 vaccine strains in eggs and purified the vNAs using the identified INAAC strategies (Fig. 5*A*). As expected, most (∼60%) NA activity from the H1N1 and H3N2 viruses was recovered in the pellet following sedimentation of the allantoic fluid (Fig. 5*B*). However, ∼65% of the B virus NA (vNB) activity remained in the supernatant, suggesting either suboptimal sedimentation conditions or vNB is shed from the virus. Following the isolation, we observed distinct elution peaks for vN1 and vNB, but a negative peak followed by a smaller positive peak for vN2 (Fig. 5*C*). We recovered more than 50% of the solubilized vN1 and vNB activities, but only ∼20% of the vN2 activity, with the majority (∼75%) found in the FT, indicating inefficient capture (Fig. 5*D*). Based on a previous study of the MAb (12), we speculate that the larger, more complex *N-*linked glycans added by avian cells reduced the vN2 capture efficiency compared to rfN2.

Despite the differences, we obtained sufficient amounts of all three vNAs with the expected molecular weights (Fig. 5*E* and Table S1). For vNB, we also observed a faster migrating band that reacted with sera raised against secreted recombinant NB (Fig. S6). Based on this immunoblot result and the amount of vNB activity shed by the virus, we interpreted the lower band as vNB head domains that result from proteolysis in the stalk. Under NR conditions, vN1 was resolved as monomers, dimers, and SDS-resistant tetramers (Fig. 5*E*), vN2 showed only dimers and tetramers, whereas vNB primarily migrated as monomers and dimers despite having a conserved stalk cysteine at position 54. The unexpected observations of monomers and partially cleaved vNB suggest that viruses may carry NAs with variable conformations.

Kinetic analysis at pH 7 revealed that vNB possesses the highest affinity to MUNANA (lowest *K*_*m*_), followed by vN1 and vN2, while vN2 had the highest catalytic rate (*V*_*max*_) (Fig. 5*F*). However, it is possible that the functional NA fraction in each preparation varies, contributing to the observed *V*_*max*_ differences (27, 51, 55). All three vNAs cleaved both α2−3-linked and α2−6-linked sialic acids (Fig. S7) and there was an indication that vNB has a higher relative capacity to cleave α2−6-linked sialic acids than vN1 and vN2 (Fig. 5*G*). With an immobilized multivalent glycoprotein substrate, vNB showed the highest specific activity followed by vN1, while vN2 exhibited poor activity (Fig. 5*H*), supporting that recent N2 activities on multivalent substrates are highly dependent on HA-mediated binding (55). Altogether, these data support that INAAC can be used for isolating and characterizing enzymatically active full-length NAs from recent H1N1, H3N2 or type B influenza viruses or recombinant sources.

## Discussion

Vaccines against envelope viruses commonly target surface proteins that are integrated into the viral membrane. Two common challenges for these types of vaccines are the need for strategies to efficiently manufacture the viral membrane proteins in their native conformation and measure the amount or potency to ensure dose consistency. For influenza vaccines, strategies have been established for producing and measuring HA antigens, but not NA antigens. The latter has hindered the development of vaccines capable of eliciting protective responses against both HA and NA for several decades. In this study we report the development of an Influenza NA Active-site Affinity Chromatography (INAAC) strategy for isolating and characterizing native NA antigens from influenza A and B viruses. Our results showed that INAAC successfully isolated enzymatically active full-length NAs from recent vaccine viruses (H1N1, H3N2 and type B) propagated in eggs or recombinant sources. Analysis by ELISA and cryo-EM showed that the NAs isolated from viruses can be used for quantifying NA content in commercial vaccines and for structural studies of entire NA antigens. These results support the notion that this simple procedure for isolating unmodified NAs can help to manufacture NA vaccine antigens and standards, and to obtain structural information of entire NAs isolated from viruses.

Our initial goal was to develop a process for isolating NAs from a variety of sources including viruses to aid in the characterization and production of vaccine antigens and standards. To minimize strain change impacts, we focused on creating an affinity-based isolation strategy that targets the conserved NA sialic acid-binding site. As a proof-of-concept, we used a recently identified MAb (FNI-9) that mimics the interaction of sialic acid with a broad array of NAs (12). Based on initial screens with the NA from H1N1, we covalently attached the MAb to beads by the *N-*linked glycans because the capture efficiency was higher than attachment *via* free amino groups. While the approach was successful for capturing NAs from an H1N1 and type B strain, the original MAb was a poor ligand for the H3N2 strain. Therefore, we used a structurally guided point substitution, R27D in the MAb light chain, which increases the affinity for N2 by replacing a charge clash with a complementary charge (30), and changed to a pH 8 buffer as this further increased capture. Although these changes improved N2 capture, further improvement by additional modifications or ligand exchange are likely warranted.

Initial elution trials for the three INAAC-captured full-length NAs (N1, N2, and NB) were conducted using both CaCl_2_ and Zana. Although both agents were effective, Zana resulted in broader peaks and was difficult to remove *via* dialysis, likely due to a slow dissociation rate from the NA active site. Consequently, we optimized the CaCl_2_ conditions to ensure recovery of enzymatically active NA. In general, we determined the minimum CaCl_2_ concentration required for efficient elution by monitoring conductance, absorbance, and NA activity. This process identified minimum CaCl_2_ concentrations of 1.25 M for vN1, 0.75 M for vN2, and 1.0 M for vNB. However, these concentrations may require re-optimization for NAs from different viral strains, particularly those with substitutions near the active site. Stability assessments revealed that all three NA activities were unaffected by prolonged exposure to CaCl_2_ concentrations up to 375 mM and that the impact at higher concentrations is pH dependent. These findings supported the buffer pH selection and the collection strategy of pre-loading collection tubes with two fraction volumes of Ca^2+^-free buffer to immediately dilute the CaCl_2_, minimizing exposure to high ionic strength conditions.

Analysis of the isolated NA led to two surprising observations. One was the presence of what we interpreted as improperly assembled N1 in viruses and the other was the prevalence of proteolytically cleaved NA in the type B virus, which both suggest viruses carry NAs in different oligomeric (assembled) states. We reached this conclusion for vN1 based on the observations of monomeric forms on nonreducing gels that were not observed with rfN1 and a lower *V*_*max*_, despite using an almost identical TX100 isolation strategy. In addition, the same vN1 isolated with the TDM strategy had a *V*_*max*_ ∼20% lower than the TX100-isolated vN1, suggesting different virus propagations may possess variable amounts of functional NA. We speculate that the potential heterogeneity in vN1 structural states could serve as an immune evasion strategy, similar to reports indicating the envelope glycoprotein 160 exists in different conformational states in HIV (64, 65, 66). Importantly, it also raises the question of whether viral-based influenza vaccines possess NA antigens in different states, and if so, could the efficacy be improved by separating them. For vNB, it is not clear if the proposed cleavage applies to NAs from other type B strains, but this could be a mechanism to create decoy antigens or adjust the functional balance of NA and HA. Future studies could use INAAC to isolate NAs from commercial vaccines for structural and immunogenicity analyses to address these critical questions.

From a structural perspective, the cryo-EM data show that the full-length NAs, purified in detergent solutions, are structurally intact. This supports that INAAC offers several advantages over current approaches for isolating NAs, including applications for characterizing NAs isolated directly from field strains and for performing more in-depth characterizations of the stalk and transmembrane domains. The initial vN1 images provide the first glimpse of these potential benefits by revealing that the stalk is asymmetrically positioned relative to the enzymatic head domain. It also suggests that the two domains are connected by a flexible linker. We speculate this linker functions as a hinge, enabling the head to tilt, similar to what was predicted in a previous computational study (67). Compared to a rigid structure, a hinge would expand substrate access with respect to the membrane, and our structure suggests the tilt angle may be restricted by steric interactions between the *N-*linked glycans on the stalk and the bottom of the head. While additional studies are needed to confirm the existence of a hinge, it is equally interesting to see if the stalk provides different functional advantages or restrictions to other NAs, like N2 and type B.

In summary, INAAC is a simple procedure for isolating nonmodified full-length NAs from a variety of sources with minimal strain-specific modifications. The largest modification was the requirement of two different ligands for isolating NAs from the H1N1, H3N2 and type B influenza vaccine viruses shown here. Overall, our results support that INAAC is suitable for isolating NA vaccine antigens, producing appropriate standards to quantify NA content in viral-based vaccines, and obtaining structural information on full-length NA antigens isolated directly from viruses. In time we expect that the INAAC strategy described here will continue to improve due to the high number of novel NA ligands being identified and that it will ultimately aid in the structural engineering and validation of NA vaccine antigens. Finally, with the expanding repertoire of available antibodies against membrane proteins, this strategy has the potential to decrease the time needed for developing vaccines based on specific antigens as well as biologics that target cellular membrane proteins.

## Experimental procedures

### Design and expression of the NA active-site-binding antibodies

NA active-site-binding antibodies were generated as previously described (30). Briefly, variable regions of the heavy (V_H_) and light (V_L_) chains from the reported human antibody FNI-9 (12) were inserted into the murine IgG1 heavy chain (CH1 + Hinge + CH2 + CH3 regions) and light chain (kappa), respectively. Both genes were synthesized with the murine IgG heavy chain signal peptide after codon optimization and inserted into pcDNA3.4 using *Eco*RI and *Hind*III sites. Antibodies were expressed by transfecting Expi293 cells with the resulting plasmids and isolated from the clarified Expi293 expression medium (4000×*g*; 10 min) by Protein A agarose (G-Biosciences) according to the manufacturer’s protocol.

### INAAC resin and column preparation

A GlycoLink Immobilization Kit (Thermo Scientific) was used to attach antibodies (∼9 mg) to hydrazide-activated agarose beads. Following the manufacturer’s instructions, antibodies were treated with sodium periodate and coupled to ∼1 ml resin in the presence of sodium cyanoborohydride. Unreacted sites were quenched, and the resin was washed with 1 M NaCl. Resin was resuspended with wash buffer (20 mM HEPES pH 7, 150 mM NaCl, 1 mM CaCl_2_, and 0.025% TX100), packed into a 1 ml AKTA Start compatible column and stored at 4 °C for use.

### Baculovirus expression of recombinant full-length NAs (rfNAs) in insect cells

Baculoviruses (BVs) expressing rfNAs were generated using the Bac-to-Bac BV Expression system (Gibco). Briefly, full-length NAs from the H1N1 vaccine strain A/Victoria/2570/2019 (EPI1741925), H3N2 strain A/Darwin/9/2021 (EPI1888081) and type B strain B/Austria/1359417/2021 (EPI1924335) were codon optimized for insect cells, synthesized and inserted into the pFastBac1 vector *Not*I and *Hind*III sites (GenScript). Plasmids were used to transform DH10Bac *E. coli* cells. Positive clones, identified by blue-white and PCR screening, were grown, and DNA was isolated with a BAC DNA miniprep kit (Zymo Research). P0, P1 and working P2 BV stocks were generated as previously described (68). *Spodoptera frugiperda* 9 (*Sf*9) cells grown at 27 °C in 0.3 l of Sf-900 III SFM (Thermo Fisher Scientific) to a density of ∼4 × 10^6^ cells/ml were infected with the P2 BV (2% v/v). Cells were harvested 72 h post-infection by sedimentation (4000×*g*; 10 min) and cell pellets were stored at −80 °C.

### INAAC optimization using rfN1 produced in insect cells

Pellets of *Sf*9 cells expressing rfN1 were solubilized overnight at 4 °C in 200 ml of N1 TX100 lysis buffer (20 mM HEPES pH 7.0, 300 mM NaCl, 1 mM CaCl_2_, and 2% TX100) supplemented 10 units/ml Benzonase (MilliporeSigma) and 1X protease inhibitor (SIGMAFAST EDTA-free Protease Inhibitor Tablets). The solubilized lysate was clarified by sedimentation (100,000×*g*; 1 h) and passed through a 0.22 μm filter. For each trial, clarified lysate (40 ml) was loaded onto a 1 ml INAAC column containing FNI-9 at 1 ml/min using an AKTA start. Columns were washed with 25 column volumes (CVs) of N1 wash buffer (20 mM HEPES pH 7.0, 150 mM NaCl, 1 mM CaCl_2_, and 0.025% TX100) and the rfN1 was eluted using 0.1 M Gly buffer pH 2.5 containing 1 mM CaCl_2_ and 0.025% TX100, or wash buffer containing 3 M CaCl_2_ or 200 μM zanamivir. Absorbance (A) at 280 nm and conductivity were monitored throughout. NA activity in fixed volumes of lysate, flow-through and fractions were analyzed using 2′-(4-methylumbelliferyl)-α-D-*N*-acetylneuraminic acid (MUNANA). Fractions with A_280nm_ above background were pooled, concentrated with a 100 kDa molecular weight cut-off (MWCO) centrifugal filter (Amicon), and dialyzed at 4 °C against 1 L wash buffer in a 3.5-kDa MWCO slide-a-lyzer (Thermo Fisher Scientific). Protein concentration was determined by a Microplate BCA assay using BSA as a standard (Pierce).

### Virus propagation and isolation by sedimentation

The following recommended egg-based vaccine strains (2022-23 season) were kindly provided by Professor Ian Barr (WHO Collaborating Center - Melbourne, Australia): B/Austria/1359417/2021, A/Victoria/2570/2019 (H1N1) and A/Darwin/9/2021 (H3N2). Viruses were diluted 1:1000 in sterile PBS pH 7.2, 0.1 ml was injected to 10-day-old SPF chicken embryonic eggs. Eggs were incubated at 33 °C for 3 days and transferred to 4 °C for 16 h. Allantoic fluid was harvested and clarified by centrifugation (1000×*g*; 10 min) at 4 °C. Clarified allantoic fluid (28 ml) was transferred to Ultraclear Beckman centrifuge tubes and underlayed with a 4 ml Sucrose cushion (PBS pH 7.2 containing 25% (w/v) sucrose and 1 mM CaCl_2_). Viruses were sedimented at 4 °C using a SW32Ti swinging bucket (100,000×*g*; 45 min), supernatant was retained for analysis, and virus pellets were resuspended directly in lysis buffer for vNA purification.

### INAAC isolation of TX100 solubilized vN1 from egg propagated viruses

vN1 isolation using TX100 was similar to rfN1. Sedimented egg-propagated H1N1 virus was solubilized overnight at 4 °C in TX100 lysis buffer containing 10 units/ml Benzonase and 1X protease inhibitor (1/10 initial allantoic fluid volume). The solubilized viral lysate was clarified by sedimentation, loaded onto a 1 ml INAAC column, washed as described for rfN1, and the vN1 was step eluted using wash buffer with 1.25 M CaCl_2_. Measurements and post-elution processing of vN1 were identical to rfN1.

### INAAC isolation of NAs using maltoside detergents

INAAC isolations with maltoside detergents (Anatrace) were carried out with the following modifications for either *Sf*9 cell pellets or sedimented egg-propagated viruses. For rfN1 or vN1 isolation with *n*-hexadecyl-β-D-maltoside (HDM), pellets (cells/viruses) were solubilized with N1 TX100 lysis buffer and applied to the column. Column was washed with N1 HDM wash buffer (20 mM HEPES pH 7, 150 mM NaCl, 1 mM CaCl_2_, and 0.0001% HDM) and eluted with N1 HDM wash buffer containing 1.25 M CaCl_2_. rfNB, rfN2, vNB and vN2 isolated with *n*-tetradecyl-β-D-maltoside (TDM), pellets (cells/viruses) were solubilized with NB/N2 TX100 lysis buffer (25 mM Tris-HCl pH 8.0, 300 mM NaCl, 1 mM CaCl_2_, and 2% TX100) and applied to the column. Column was washed with NB/N2 TDM wash buffer (25 mM Tris pH 8.0, 150 mM NaCl, 1 mM CaCl_2_, and 0.015% TDM) and eluted with wash buffer containing either 1 M CaCl_2_ (NB), 0.75 M CaCl_2_ (N2) or 1 mM zanamivir (NB and N2). N2 changes also included an INAAC column containing the R27D modified FNI-9 described previously. vN1 isolated with TDM was performed as described above, with the exchange of 0.015% TDM for 0.0001% HDM. All CaCl_2_ elution fractions with TDM were rapidly diluted 1:3 by using tubes containing two fraction volumes of N1 or NB/N2 wash buffer without detergent. Peak fractions were pooled, concentrated ∼10-fold and exchanged into HBS (20 mM HEPES pH 7.0, 150 mM NaCl, and 1 mM CaCl_2_) using 100 kDa MWCO centrifugal filters.

### SDS-PAGE analysis, immunoblotting, and protein purity determination

Purified NAs (∼2 μg) were mixed with equal volume of 2X Tris-Glycine SDS sample buffer with or without 100 mM dithiothreitol (DTT) and heated either at 50 °C or at 95 °C for 5 min prior to loading on 8% to 16% or 4 to 12% Novex Tris-Glycine SDS-PAGE WedgeWell gels (Thermo Fisher Scientific). Gels were run at constant voltage (150V) for 1 h, washed with dH_2_O (3 times, 5 min), stained with Simply blue (Thermo Fisher Scientific) and visualized with an Azure 600 (Azure Biosystems). Novex Sharp Unstained protein standard (Thermo Fisher Scientific) was included for molecular weight references. For immunoblotting, proteins were resolved by reducing SDS-PAGE (+DTT) and transferred to a 0.22 μm nitrocellulose membrane. Membranes were blocked with PBS containing 0.05% Tween 20 (PBST) and 5% BSA, probed with mouse antisera raised against heat denatured recombinant NB overnight at 4 °C, washed 5 times for 10 min with PBST, incubated with a horse radish peroxidase labeled anti-mouse IgG secondary for 1 h at 37 °C, and visualized with SuperSignal West Dura Extended Duration Substrate (Thermo Fisher Scientific) using an Azure 600. All images were converted to gray scale with Adobe Photoshop 2023, and immunoblot images were inverted. Protein purity was determined by densitometry analysis of the Coomassie stained gel using ImageJ. Briefly, density of the band corresponding to NA monomers of the expected size was divided by the total density in the lane and multiplied by 100%.

### NA activity and kinetic analysis with MUNANA

Sialidase activity measured with MUNANA was performed as previously described (51). Briefly, samples (10 μl) containing NA or the indicated amounts of isolated NA were added to 96-well, black wall, clear bottom plates (Corning). Reactions were initiated by adding 90 μl of 37 °C substrate solution (5 μl of 2 mM MUNANA and 85 μl of 0.1 M Tris-HCl pH 7 containing 150 mM NaCl and 1 mM CaCl_2_) to each well. Reactions at 37 °C were read by measuring the fluorescence (λEx: 355 nm, λEm: 450 nm) for 10 min at 30 s intervals on a Cytation 5 Plate Reader (Biotek). Michaelis-Menten kinetic analyses were performed similarly, except the indicated isolated NA amounts were added to wells containing two-fold serial dilutions of MUNANA with final concentrations that ranged from 2.81 to 360 μM. Activities were reported as RFU/sec determined using the initial linear readings or transformed into pmol/sec using a 4-methylumbelliferone standard curve. *K*_*m*_ and *V*_*max*_ values were determined by a nonlinear regression analysis in GraphPad Prism 10 software.

### Synthesis of Neu5Acα2–6GalβMU

GalβMU (100 mg, 0.30 mmol), CTP (234 mg, 0.44 mmol), and Neu5Ac (119 mg, 0.38 mmol) were dissolved in water in a 50 ml centrifuge tube containing Tris-HCl buffer (100 mM, pH 8.5) and MgCl_2_ (20 mM). Recombinant *Neisseria meningitidis* CMP-sialic acid synthetase (69) (3 mg) and a *Photobacterium* sp. α2–6-sialyltransferase mutant (70) (3 mg) were added. The reaction mixture (20 ml) was incubated at 30 °C with agitation at 180 rpm. Product formation was monitored by high-resolution mass spectrometry (HRMS). After 16 h, enzymes in the reaction mixture were denatured by incubation in a boiling water bath for 5 min and removed by centrifugation (10,000×*g*; 30 min) at 4 °C. The supernatant was concentrated and purified by a preconditioned DSC-18 SPE cartridge (bed weight 10 g). After washing the cartridge with water (30 ml), a mixture solvent of methanol in water (40%) was used to elute the product. Solvent was removed by evaporation *in vacuo* and the pure product was obtained as a white powder (164 mg, 85% yield). ^1^H NMR (D_2_O, 400 MHz) δ 7.86–7.71 (m, 1H), 7.21–7.16 (m, 2H), 6.30 (d, *J* = 1.2 Hz, 1H), 5.16 (d, *J* = 7.6 Hz, 1H), 4.16–3.41 (m, 13H),i NMR (D_2_O, 100 MHz) δ 174.98, 173.52, 164.29, 159.55, 156.03, 153.63, 126.52, 114.93, 113.92, 111.11, 103.56, 100.18, 74.01, 72.56, 72.39, 71.67, 70.38, 68.41, 68.23, 68.16, 63.00, 62.55, 51.82, 40.49, 21.96, and 18.01. HRMS (ESI/Orbitrap) m/z: [M-H]^-^ calculated for C_27_H_34_NO_16_ 628.1883; found 628.1880.

### NA substrate specificity assays

Specificity assays for α2–3-linked and α2–6-linked sialic acid were performed using Neu5Acα2–3GalβMU or Neu5Acα2–6GalβMU synthesized as previously described (52). Initially, vNAs were standardized based on activity using Neu5Acα2–3GalβMU with a final volume of 80 μl in 96-well, black wall, clear-bottom plates. vNAs were serially diluted and added (10 μl) to wells containing 70 μl of 37 °C substrate solution (2 μl of 1 mM Neu5Acα2–3GalβMU, 2 μl of 6 mg/ml galactosidase (Sigma) and 66 μl of 25 mM MES pH 7 with 150 mM NaCl and 1 mM CaCl_2_). Fluorescence (λEx: 355 nm, λEm: 450 nm) at 37 °C was measured (30 min; 30 s intervals) on a Cytation 5 Plate Reader. Activities were determined using the linear reading from 5 to 20 min and amounts were normalized using the slope ratios. Following normalization, three independent activity assays were carried out for each protein and substrate using two-fold serial dilutions as described. Reactions without NA were used as negative controls and for background readings.

### ELISA analysis of NA content in commercial vaccines

Immulon 4HB 96-well microtiter plates (Thermo Fisher Scientific) were coated with the recombinant FNI-9 antibody (0.5 μg/well) described above in phosphate-buffered saline (PBS) pH 7.4 overnight at 4 °C. Wells were blocked with 200 μl of 3.0% Bovine serum albumin (BSA) (Sigma) in PBS pH 7.4 for 1 h at 37 °C. Blocking solution was removed and 2-fold serial dilutions of vN1, rfN1 and commercial vaccine in dilution buffer (20 mM HEPES pH 7.0, 300 mM NaCl, 1 mM CaCl_2_, and 0.5% BSA) were transferred in duplicate to the plate. The plate was incubated at 37 °C for 2 h, washed (6 × 200 μl) with washing buffer (PBS pH 7.4 containing 0.025% TX100) and incubated at 37 °C for 2 h with an HRP-conjugated N1-specific mAb 5D11 diluted to ∼0.5 μg/ml in dilution buffer (30). Plates were washed with washing buffer (3 × 200 μl) and developed with OPD for 10 min at 37 °C. Reactions were stopped with 1 N H_2_SO_4_, and the A_490nm_ was read with a Cytation five plate reader. A_490nm_ results were plotted in GraphPad Prism 10 software were used to calculate the vN1 4-Parameter Logistic (4 PL) regression equation used to determine the NA amount in the vaccine.

### Cryo-EM sample preparation and data acquisition

vN1 protein dialyzed into N1 HDM wash buffer was concentrated to 1.7 mg/ml and stored at −80 °C until processing. Upon thawing, the protein remained on ice prior to freezing. For the vN1-zana sample, the vN1 was adjusted to 10 μM zanamivir and incubated ∼1 h prior to freezing. The holey-carbon cryo-EM grids (Quantifoil, Au R1.2/1.3, 300 mesh) were glow-discharged at 5 mA for 30s. A volume of 2.8 μl of the sample was applied on the grids, grids were blotted for 4s with blot force set to 0 in a Vitrobot Mark IV (ThermoFisher) at 15 °C and 100% humidity, and plunge frozen using a liquid ethane-propane mixture. Grids were stored in liquid nitrogen until imaging. Cryo-EM data were collected on a 200-keV Talos Arctica microscope at the University of Groningen equipped with a BioQuantum K2 (Gatan) using a 20-eV energy slit and a 100 μm objective aperture. Data was recorded using EPU software (v3.2) in the counting mode at the nominal magnification of 130,000 (pixel size of 1.022 Å) with applied defocus between −0.9 and −1.9 μm. 9 s exposures were taken in each hole, positioned slightly off-center, collecting 60 frames with a total dose of approximately 53 to 57 electrons per Å^2^. The hole targets with ice thickness ranging from 20 to 50 nm were selected based on optimizing the Filter Ice Quality values, which was guided by the estimated ice thickness calculated in Digital Micrograph using an in-house written script (71).

### Cryo-EM image processing

Datasets collected for vN1 and vN1-zana samples were processed using the same workflow. Movies were preprocessed in WARP [v1.0.9 (72)], which included motion correction and CTF estimation. Micrographs were manually inspected, and those with suboptimal parameters defined by defocus values < 0.5 μm or > 2 μm, resolution < 4.5 Å, or visible ice contamination, were discarded. Particles picking was performed in crYOLO [v1.7.6 (73)] using the general model. The selected particles were imported into Relion (v3.1.4) (74) and extracted with a box size of 300 pixels (1.044 Å pix^-1^). Extracted particles were imported to cryoSPARC [v4.5.1 (75)] for a round of 2D classification (100 classes). 2D classes displaying clear protein features were selected and used to generate three initial *ab initio* volumes, which served as input for heterogeneous refinement to separate particles. Particles from the best resolved class were refined using non-uniform refinement and subsequently converted to a star file for re-extraction in Relion (v3.1.4) with a box size of 256 pix (1.022 Å pix^−1^). Selected particles underwent iterative rounds of 3D refinement, CTF refinement, and Bayesian polishing (76). Final 3D refinement was performed in Relion (v5) using blush regularization, both with and without applying C4 symmetry. Local resolution was estimated in Relion (v5).

### Model building and refinement

Final unsharpened maps were used for model building and refinement. The previously published structure of N1 [PDB 3NSS (50)] was used as a reference for model building and initially adjusted in Coot [v0.9 (77)]. Models were iteratively adjusted using Coot (v0.9) and ISOLDE [v1.9 (78)], followed by real-space refinement in Phenix [v1.21.2-5419 (79)]. DeepEMhancer maps were used for figure preparation. Figures were prepared in ChimeraX [v1.9 (80)]. All software used for image processing and model refinement was accessed through SBGrid (81).

### Statistics and reproducibility

GraphPad Prism 10 software was used to perform Student’s unpaired t-tests, linear and the nonlinear 4-parameter logistic regression analyses. Student’s unpaired t-tests were performed using a 95% confidence interval (CI) with the assumption that data from three or more independent biological replicates follow a Gaussian distribution with equal standard deviation between sample groups. Linearity was assessed by comparing the slope ± 95% CI of the log-log linear regression equation. *p* values <0.05 were considered significant.

## Ethics statement

The contents of this publication are an informal communication and represent the best judgment of the authors. These comments do not bind or obligate FDA.

## Data availability

All datasets are presented in the manuscript and supplemental material. Cryo-EM density maps, half maps, and masks have been deposited in the Electron Microscopy Data Bank (EMDB), and the atomic models are available through the Protein Data Bank (PDB). The data are available under the following accession codes: vN1 (PDB ID 9TQ7, EMD-56126) and vN1 in complex with zanamivir (PDB ID 9TQ8, EMD-56127).

## Supporting information

This article contains supporting information (75, 82).

## Conflict of interest

The authors declare the following financial interests/personal relationships, which may be considered as potential competing interests: H. Y. and X. C. collaborate with Integrated Micro-Chromatography System (IMCS) on projects funded by the National Institutes of Health (NIH) (including grant number: R42GM143998). IMCS played no role in the design, execution, interpretation, or publication of this study.

## References

[1] Altman M.O., Angel M., Kosik I., Trovao N.S., Zost S.J., Gibbs J.S. (2019). Human influenza A virus Hemagglutinin Glycan Evolution follows a temporal pattern to a Glycan limit. mBio.

[2] Dou D., Revol R., Östbye H., Wang H., Daniels R. (2018). Influenza A virus cell entry, replication, virion assembly and movement. Front Immunol..

[3] Suzuki Y. (2005). Sialobiology of influenza: molecular mechanism of host range variation of influenza viruses. Biol. Pharm. Bull.

[4] Weis W., Brown J.H., Cusack S., Paulson J.C., Skehel J.J., Wiley D.C. (1988). Structure of the influenza virus haemagglutinin complexed with its receptor, sialic acid. Nature.

[5] Matrosovich M., Herrler G., Klenk H.D. (2015). Sialic acid receptors of viruses. Top Curr. Chem..

[6] Hirst G.K. (1942). Adsorption of influenza hemagglutinins and virus by red blood cells. J. Exp. Med..

[7] Gottschalk A. (1957). Neuraminidase: the specific enzyme of influenza virus and Vibrio cholerae. Biochim. Biophys. Acta.

[8] Kilbourne E.D., Laver W.G., Schulman J.L., Webster R.G. (1968). Antiviral activity of antiserum specific for an influenza virus neuraminidase. J. Virol..

[9] Palese P., Compans R.W. (1976). Inhibition of influenza virus replication in tissue culture by 2-deoxy-2,3-dehydro-N-trifluoroacetylneuraminic acid (FANA): mechanism of action. J. Gen. Virol..

[10] Wei C.J., Crank M.C., Shiver J., Graham B.S., Mascola J.R., Nabel G.J. (2020). Next-generation influenza vaccines: opportunities and challenges. Nat. Rev. Drug Discov..

[11] Murphy B.R., Kasel J.A., Chanock R.M. (1972). Association of serum anti-neuraminidase antibody with resistance to influenza in man. N. Engl. J. Med..

[12] Momont C., Dang H.V., Zatta F., Hauser K., Wang C., di Iulio J. (2023). A pan-influenza antibody inhibiting neuraminidase via receptor mimicry. Nature.

[13] Stadlbauer D., Zhu X., McMahon M., Turner J.S., Wohlbold T.J., Schmitz A.J. (2019). Broadly protective human antibodies that target the active site of influenza virus neuraminidase. Science.

[14] Yasuhara A., Yamayoshi S., Kiso M., Sakai-Tagawa Y., Okuda M., Kawaoka Y. (2022). A broadly protective human monoclonal antibody targeting the sialidase activity of influenza A and B virus neuraminidases. Nat. Commun..

[15] Francis T., Salk J.E., Pearson H.E., Brown P.N. (1945). Protective effect of vaccination against induced influenza A. J. Clin. Invest..

[16] Salk J.E., Pearson H.E., Brown P.N., Francis T. (1945). Protective effect of vaccination against induced influenza B. J. Clin. Invest..

[17] Hirst G.K. (1942). The quantitative determination of influenza virus and antibodies by means of red cell agglutination. J. Exp. Med..

[18] Hirst G.K., Rickard E.R., Whitman L., Horsfall F.L. (1942). Antibody response of human beings following vaccination with influenza viruses. J. Exp. Med..

[19] Weir J.P., Gruber M.F. (2016). An overview of the regulation of influenza vaccines in the United States. Influenza Other Respir. Viruses.

[20] Brand C.M., Skehel J.J. (1972). Crystalline antigen from the influenza virus envelope. Nat. New Biol..

[21] Catani J.P.P., Smet A., Ysenbaert T., Vuylsteke M., Bottu G., Mathys J. (2024). The antigenic landscape of human influenza N2 neuraminidases from 2009 until 2017. Elife.

[22] Smith D.J., Lapedes A.S., de Jong J.C., Bestebroer T.M., Rimmelzwaan G.F., Osterhaus A.D. (2004). Mapping the antigenic and genetic evolution of influenza virus. Science.

[23] Gao J., Li X., Klenow L., Malik T., Wan H., Ye Z. (2022). Antigenic comparison of the neuraminidases from recent influenza A vaccine viruses and 2019-2020 circulating strains. NPJ Vaccin..

[24] Bedford T., Suchard M.A., Lemey P., Dudas G., Gregory V., Hay A.J. (2014). Integrating influenza antigenic dynamics with molecular evolution. Elife.

[25] Johansson B.E., Matthews J.T., Kilbourne E.D. (1998). Supplementation of conventional influenza A vaccine with purified viral neuraminidase results in a balanced and broadened immune response. Vaccine.

[26] Kang H., Martinez M.R., Aves K.L., Okholm A.K., Wan H., Chabot S. (2024). Capsid virus-like particle display improves recombinant influenza neuraminidase antigen stability and immunogenicity in mice. iScience.

[27] Gao J., Klenow L., Parsons L., Malik T., Phue J.N., Gao Z. (2021). Design of the recombinant influenza neuraminidase antigen is crucial for its biochemical properties and protective efficacy. J. Virol..

[28] Ellis D., Lederhofer J., Acton O.J., Tsybovsky Y., Kephart S., Yap C. (2022). Structure-based design of stabilized recombinant influenza neuraminidase tetramers. Nat. Commun..

[29] Strohmeier S., Carreno J.M., Brito R.N., Krammer F. (2021). Introduction of cysteines in the stalk domain of recombinant influenza virus N1 neuraminidase enhances protein stability and immunogenicity in mice. Vaccines (Basel).

[30] Gao J., Landgraf G., Yuan Y., Yu H., Saeidi S., Kang H. (2025). Influenza neuraminidase active site proximity assay for rapid profiling of inhibitory antibodies and antigenic drift. NPJ Vaccin..

[31] Couzens L., Gao J., Westgeest K., Sandbulte M., Lugovtsev V., Fouchier R. (2014). An optimized enzyme-linked lectin assay to measure influenza A virus neuraminidase inhibition antibody titers in human sera. J. Virol. Methods.

[32] Gao J., Couzens L., Eichelberger M.C. (2016). Measuring influenza neuraminidase inhibition antibody titers by enzyme-linked lectin assay. J. Vis. Exp..

[33] Cate T.R., Rayford Y., Nino D., Winokur P., Brady R., Belshe R. (2010). A high dosage influenza vaccine induced significantly more neuraminidase antibody than standard vaccine among elderly subjects. Vaccine.

[34] Fritz R., Sabarth N., Kiermayr S., Hohenadl C., Howard M.K., Ilk R. (2012). A vero cell-derived whole-virus H5N1 vaccine effectively induces neuraminidase-inhibiting antibodies. J. Infect Dis..

[35] Sandbulte M.R., Gao J., Straight T.M., Eichelberger M.C. (2009). A miniaturized assay for influenza neuraminidase-inhibiting antibodies utilizing reverse genetics-derived antigens Influenza. Other Respir. Viruses.

[36] Sultana I., Yang K., Getie-Kebtie M., Couzens L., Markoff L., Alterman M. (2014). Stability of neuraminidase in inactivated influenza vaccines. Vaccine.

[37] Byrne-Nash R.T., Gillis J.H., Miller D.F., Bueter K.M., Kuck L.R., Rowlen K.L. (2019). A neuraminidase potency assay for quantitative assessment of neuraminidase in influenza vaccines. NPJ Vaccin..

[38] Gao Z., Robinson K., Skowronski D.M., De Serres G., Withers S.G. (2020). Quantification of the total neuraminidase content of recent commercially-available influenza vaccines: introducing a neuraminidase titration reagent. Vaccine.

[39] Varghese J.N., Laver W.G., Colman P.M. (1983). Structure of the influenza virus glycoprotein antigen neuraminidase at 2.9 A resolution. Nature.

[40] Wang H., Dou D., Östbye H., Revol R., Daniels R. (2019). Structural restrictions for influenza neuraminidase activity promote adaptation and diversification. Nat. Microbiol..

[41] Gao J., Wan H., Li X., Rakic Martinez M., Klenow L., Gao Y. (2021). Balancing the influenza neuraminidase and hemagglutinin responses by exchanging the vaccine virus backbone. Plos Pathog..

[42] Harris A., Cardone G., Winkler D.C., Heymann J.B., Brecher M., White J.M. (2006). Influenza virus pleiomorphy characterized by cryoelectron tomography. Proc. Natl. Acad. Sci. U S A..

[43] Wang N., Glidden E.J., Murphy S.R., Pearse B.R., Hebert D.N. (2008). The cotranslational maturation program for the type II membrane glycoprotein influenza neuraminidase. J. Biol. Chem..

[44] Nordholm J., da Silva D.V., Damjanovic J., Dou D., Daniels R. (2013). Polar residues and their positional context dictate the transmembrane domain interactions of influenza A neuraminidases. J. Biol. Chem..

[45] da Silva D.V., Nordholm J., Madjo U., Pfeiffer A., Daniels R. (2013). Assembly of subtype 1 influenza neuraminidase is driven by both the transmembrane and head domains. J. Biol. Chem..

[46] da Silva D.V., Nordholm J., Dou D., Wang H., Rossman J.S., Daniels R. (2015). The influenza virus neuraminidase protein transmembrane and head domains have coevolved. J. Virol..

[47] Colman P.M., Varghese J.N., Laver W.G. (1983). Structure of the catalytic and antigenic sites in influenza virus neuraminidase. Nature.

[48] Laver W.G. (1978). Crystallization and peptide maps of neuraminidase "heads" from H2N2 and H3N2 influenza virus strains. Virology.

[49] Strohmeier S., Amanat F., Zhu X., McMahon M., Deming M.E., Pasetti M.F. (2021). A novel recombinant influenza virus neuraminidase vaccine candidate stabilized by a measles virus phosphoprotein tetramerization domain provides robust protection from virus challenge in the mouse model. mBio.

[50] Li Q., Qi J., Zhang W., Vavricka C.J., Shi Y., Wei J. (2010). The 2009 pandemic H1N1 neuraminidase N1 lacks the 150-cavity in its active site. Nat. Struct. Mol. Biol..

[51] Kang H., Malik T., Daniels R. (2023). Isolation by multistep chromatography improves the consistency of secreted recombinant influenza neuraminidase antigens. J. Chromatogr. B Analyt Technol. Biomed. Life Sci..

[52] Yuan Y., Yu H., Agrahari A.K., Gao J., Kang H., Daniels R. (2025). Catch, cut, or block? Versatile 4-N-Derivatized sialyl glycosides for influenza virus neuraminidase detection and purification. Angew. Chem. Int. Ed. Engl..

[53] Ikematsu H., Kawai N., Tani N., Chong Y., Bando T., Iwaki N. (2020). In vitro neuraminidase inhibitory concentration (IC50) of four neuraminidase inhibitors in the Japanese 2018-19 season: Comparison with the 2010-11 to 2017-18 seasons. J. Infect Chemother..

[54] Boyd S., Yamazaki H. (1993). Efficient and gentle elution of antibodies from an immobilized polypeptide antigen BT saturated magnesium chloride. Biotechnol. Tech..

[55] Klenow L., Elfageih R., Gao J., Wan H., Withers S.G., de Gier J.W. (2023). Influenza virus and pneumococcal neuraminidases enhance catalysis by similar yet distinct sialic acid-binding strategies. J. Biol. Chem..

[56] Wan H., Gao J., Yang H., Yang S., Harvey R., Chen Y.Q. (2019). The neuraminidase of A(H3N2) influenza viruses circulating since 2016 is antigenically distinct from the A/Hong Kong/4801/2014 vaccine strain. Nat. Microbiol..

[57] An Y., Rininger J.A., Jarvis D.L., Jing X., Ye Z., Aumiller J.J. (2013). Comparative glycomics analysis of influenza Hemagglutinin (H5N1) produced in vaccine relevant cell platforms. J. Proteome Res..

[58] Mitnaul L.J., Castrucci M.R., Murti K.G., Kawaoka Y. (1996). The cytoplasmic tail of influenza A virus neuraminidase (NA) affects NA incorporation into virions, virion morphology, and virulence in mice but is not essential for virus replication. J. Virol..

[59] Ostbye H., Gao J., Martinez M.R., Wang H., de Gier J.W., Daniels R. (2020). N-Linked glycan sites on the influenza A virus neuraminidase head domain are required for efficient viral incorporation and replication. J. Virol..

[60] York I.A., Stevens J., Alymova I.V. (2019). Influenza virus N-linked glycosylation and innate. Immunity Biosci. Rep..

[61] Saeidi S., Wan H., Kang H., Gao J., Wu W.W., Malik T. (2025). N-linked glycans on the stalk of influenza virus neuraminidase promote functional tetramer formation by compensating for local hydrophobicity. J. Virol..

[62] Collins P.J., Haire L.F., Lin Y.P., Liu J., Russell R.J., Walker P.A. (2008). Crystal structures of oseltamivir-resistant influenza virus neuraminidase mutants. Nature.

[63] Colman P.M. (2009). New antivirals and drug resistance. Annu. Rev. Biochem..

[64] Lu M., Ma X., Castillo-Menendez L.R., Gorman J., Alsahafi N., Ermel U. (2019). Associating HIV-1 envelope glycoprotein structures with states on the virus observed by smFRET. Nature.

[65] Munro J.B., Gorman J., Ma X., Zhou Z., Arthos J., Burton D.R. (2014). Conformational dynamics of single HIV-1 envelope trimers on the surface of native virions. Science.

[66] Wang Q., Finzi A., Sodroski J. (2020). The conformational States of the HIV-1 envelope glycoproteins. Trends Microbiol..

[67] Casalino L., Seitz C., Lederhofer J., Tsybovsky Y., Wilson I.A., Kanekiyo M. (2022). Breathing and tilting: mesoscale simulations illuminate Influenza Glycoprotein vulnerabilities. ACS Cent. Sci..

[68] Chabot S., Malik T., Saye L., Kosikova M., Kang H., Ye Z. (2025). Development of a recombinant membrane protein ELISA for analyzing antibody responses against SARS-CoV-2 envelope proteins. J. Biol. Chem..

[69] Yu H., Yu H., Karpel R., Chen X. (2004). Chemoenzymatic synthesis of CMP-sialic acid derivatives by a one-pot two-enzyme system: comparison of substrate flexibility of three microbial CMP-sialic acid synthetases. Bioorg. Med. Chem..

[70] Ding L., Zhao C., Qu J., Li Y., Sugiarto G., Yu H. (2015). A Photobacterium sp. alpha2-6-sialyltransferase (Psp2,6ST) mutant with an increased expression level and improved activities in sialylating Tn antigens. Carbohydr. Res..

[71] Rheinberger J., Oostergetel G., Resch G.P., Paulino C. (2021). Optimized cryo-EM data-acquisition workflow by sample-thickness determination. Acta Crystallogr. D Struct. Biol..

[72] Tegunov D., Cramer P. (2019). Real-time cryo-electron microscopy data preprocessing with Warp. Nat. Methods.

[73] Wagner T., Merino F., Stabrin M., Moriya T., Antoni C., Apelbaum A. (2019). SPHIRE-crYOLO is a fast and accurate fully automated particle picker for cryo-EM. Commun. Biol..

[74] Zivanov J., Nakane T., Forsberg B.O., Kimanius D., Hagen W.J., Lindahl E. (2018). New tools for automated high-resolution cryo-EM structure determination in RELION-3. Elife.

[75] Punjani A., Rubinstein J.L., Fleet D.J., Brubaker M.A. (2017). cryoSPARC: algorithms for rapid unsupervised cryo-EM structure determination. Nat. Methods.

[76] Zivanov J., Nakane T., Scheres S.H.W. (2019). A Bayesian approach to beam-induced motion correction in cryo-EM single-particle analysis. IUCrJ.

[77] Emsley P., Cowtan K. (2004). Coot: model-building tools for molecular graphics. Acta Crystallogr. D Biol. Crystallogr..

[78] Croll T.I. (2018). ISOLDE: a physically realistic environment for model building into low-resolution electron-density maps. Acta Crystallogr. D Struct. Biol..

[79] Adams P.D., Afonine P.V., Bunkoczi G., Chen V.B., Davis I.W., Echols N. (2010). PHENIX: a comprehensive Python-based system for macromolecular structure solution. Acta Crystallogr. D Biol. Crystallogr..

[80] Goddard T.D., Huang C.C., Meng E.C., Pettersen E.F., Couch G.S., Morris J.H. (2018). UCSF ChimeraX: meeting modern challenges in visualization and analysis. Protein Sci..

[81] Morin A., Eisenbraun B., Key J., Sanschagrin P.C., Timony M.A., Ottaviano M. (2013). Collaboration gets the most out of software. Elife.

[82] Pettersen E.F., Goddard T.D., Huang C.C., Couch G.S., Greenblatt D.M., Meng E.C. (2004). UCSF Chimera--a visualization system for exploratory research and analysis. J Comput Chem.

