# Supplementary material for: INAAC: An affinity chromatography strategy enabling characterization and quantification of influenza neuraminidase antigens in vaccines
Source: J Biol Chem. 2026 May 12;302(7):113138. doi: 10.1016/j.jbc.2026.113138 (PMC13264171; doi:10.1016/j.jbc.2026.113138)
Supplement: Supplementary Tables [file mmc1.docx]

**SUPPLEMENTARY TABLES**

**INAAC: An affinity chromatography strategy enabling characterization and quantification of influenza neuraminidase antigens in vaccines**

Hyeog Kang^1^, Anna Borowska^2^, Tapan Kanai^1^, Jin Gao^1^, Hai Yu^3^, Xi Chen^3^, Jason Gorman^1^, Dirk-Jan Slotboom^2^ and Robert Daniels^1*^

**Supplementary Tables S1-S4**

**Table S1, related to Figures 1, 2 and 5**

**Table S2, related to Figure 2**

**Table S3, related to Figure 2**

**Table S4, related to Figure 3**

**Table S1.** Characteristics of recombinant and viral NAs analyzed in this study.

| **NA** | **Influenza Strains** | ***N-*linked glycan site number (stalk/head)** | **Estimated Molecular Weight^1^** | **Purity^2^** |
| --- | --- | --- | --- | --- |
| rfN1 | A/Victoria/2570/2019 (H1N1) | 8 (5/3) | 62.3 kD | 83.6 % |
| vN1 | A/Victoria/2570/2019 (H1N1) | 8 (5/3) | 68.4 kD | 82.3 % |
| rfN2 | A/Darwin/9/2021 (H3N2) | 9 (2/7) | 63.9 kD | 95.1% |
| vN2 | A/Darwin/9/2021 (H3N2) | 9 (2/7) | 71 kD | 82.9% |
| rfNB | B/Austria/1359417/2021 | 4 (2/2) | 57.5 kD | 78.8% |
| vNB | B/Austria/1359417/2021 | 4 (2/2) | 59.6 kD | 84.3% |

^1^Determined by adding 2.1 kDa for each *N-*linked glycan site in the viral derived NAs and 1.1 kDa for each site in the recombinant insect cell produced NAs.

^2^Calculated by densitometry of reducing Coomassie-stained SDS-PAGE gels.

**Table S2.** Linearity of vN1 sample dilutions measured by ELISA

| Expected vN1 Amount (ng) | Calculated Amount (ng)^1^ | | | Accuracy (%)^2^ | Coefficient of Variation %^3^ |
| --- | --- | --- | --- | --- | --- |
| 100 | 67.7 | 67.3 | 67.4 | 67.5 | 0.30 |
| **50** | **43.3** | **44.3** | **44.4** | **88.0** | **1.1** |
| **25** | **25.1** | **25.9** | **24.3** | **100** | **2.6** |
| **12.5** | **13.8** | **14.7** | **14.5** | **115** | **2.7** |
| **6.25** | **6.72** | **6.84** | **7.14** | **110** | **2.6** |
| **3.125** | **3.01** | **3.34** | **3.16** | **101** | **4.2** |
| **1.5625** | **1.37** | **1.33** | **1.43** | **88.2** | **2.9** |

Indicated vN1 amounts were analyzed by ELISA in triplicate. Samples with 85-115% accuracy are in bold.

^1^Calculated amounts were determined using the log-log linear equation.

^2^Accuracy % was determined by: (Calculated amount/Expected amount)*100%.

^3^CV % was determined by: (Calculated amount SD/Mean Calculated amount)*100%

| Fluzone volume (µl) | Mean Calculated Amount ± SD (ng)^1^ | Mean Calculated  Amount in 0.5 ml Dose ± SD (μg) | Previous dilution Concentration % | Coefficient of Variation %^3^ | Mean N1 amount in 0.5 ml dose ± SD (µg) |
| --- | --- | --- | --- | --- | --- |
| 20 | 36.7 ± 0.88 | 0.918 ± 0.02 | N/A^2^ | 2.4 |  |
| **10** | **26.8 ± 1.38** | **1.34 ± 0.07** | **100** | **5.1** | **1.42 ± 0.1** |
| **5** | **15.3 ± 1.26** | **1.53 ± 0.13** | **114** | **8.3** |  |
| **2.5** | **7.43 ± 0.544** | **1.49 ± 0.11** | **97** | **7.3** |  |
| **1.25** | **3.27 ± 0.385** | **1.31 ± 0.15** | **87** | **12** |  |
| 0.625 | 1.09 ± 0.213 | 0.87 ± 0.17 | 67 | 20 |  |
| 0.3125 | 0.295 ± 0.219 | 0.47 ± 0.35 | 54 | 74 |  |

**Table S3.** N1 ELISA results for Fluzone 2022-23

Indicated Fluzone volumes were run in triplicate. Values for determining the N1 amount per dose (bold) were identified based on CV and previous dilution concentration calculations.

^1^Determined with the 4-PL equation for vN1 run at the same time.

^2^Sample was not analyzed due to matrix effect causing loss of linearity with vN1.

^3^CV % was determined by the formula: (Calculated amount SD/Calculated amount)*100%

**Table S4.** Cryo-EM data collection, refinement and validation statistics.

|  | vN1  (PDB 9TQ7, EMD-56126) | vN1 with Zana  (PDB 9TQ8, EMD-56127) |
| --- | --- | --- |
| **Data collection and processing** | | |
| Microscope | Thermo Fisher Talos Arctica | |
| Detector | Gatan K2 Summit | |
| Camera mode | Counting mode | |
| Voltage (kV) | 200 | |
| Nominal magnification | 130,000x | |
| Total electron exposure (e–/Å^2^) | 57.3 | 52 |
| Number of frames | 60 | |
| Defocus range (μm) | -0.5 to -2.0 | |
| Pixel size (Å) | 1.022 | |
| Data collection software | EPU v3.2 | |
| Micrographs collected | 2282 | 2889 |
| Micrographs used | 1821 | 1797 |
| Initial particle images (no.) | 456,795 | 421,625 |
| Final particle images (no.) | 204,391 | 207,640 |
| Symmetry imposed | C4 | C4 |
| Global map resolution (Å)  (FSC threshold 0.143) | 3.58 | 3.48 |
| Map resolution range (Å) | ~ 3.2 – 3.8 | ~ 3.2 – 4.0 |
| **Refinement and validation** | | |
| Initial model used (PDB code) | 3NSS | 3NSS |
| Model resolution (Å)  FSC threshold (0.5) | 3.9 | 4.0 |
| Map sharpening *B* factor (Å^2^) | -157 | -157 |
| Model composition  Chains  Non-hydrogen atoms  Protein residues  Ligands  ZMR  NAG  CA | 4  12160  1548  0  12  8 | 4  12248  1548  4  12  4 |
| *B* factors (Å^2^)  Protein (min/max/mean)  Ligand (min/max/mean) | 93.97/146.44/118.48  127.41/155.86/147.80 | 133.63/191.47/161.17  169.10/203.49/180.25 |
| R.m.s. deviations  Bond lengths (Å)  Bond angles (°) | 0.004  0.573 | 0.002  0.529 |
| Validation  MolProbity score  Clashscore  Poor rotamers (%) | 1.63  4.97  0.30 | 1.77  7.54  0.00 |
| Ramachandran plot  Favored (%)  Allowed (%)  Disallowed (%) | 94.55  5.45  0.00 | 94.81  5.19  0.00 |
