# Supplementary material for: INAAC: An affinity chromatography strategy enabling characterization and quantification of influenza neuraminidase antigens in vaccines
Source: J Biol Chem. 2026 May 12;302(7):113138. doi: 10.1016/j.jbc.2026.113138 (PMC13264171; doi:10.1016/j.jbc.2026.113138)
Supplement: Supplementary Figures [file mmc2.pdf]

SUPPLEMENTARY FIGURES

INAAC: An affinity chromatography strategy enabling characterization and quantification of influenza neuraminidase antigens in vaccines

Hyeog Kang[1], Anna Borowska[2], Tapan Kanai[1], Jin Gao[1], Hai Yu[3], Xi Chen[3], Jason Gorman[1], Dirk-Jan Slotboom[2] and Robert Daniels[1*]

Supplementary Figures S1-S7

Figure S1 and S2, related to Figure 2

Figure S3 and S4, related to Figure 3

Figure S5, related to Figure 4

Figure S6 and S7, related to Figure 5

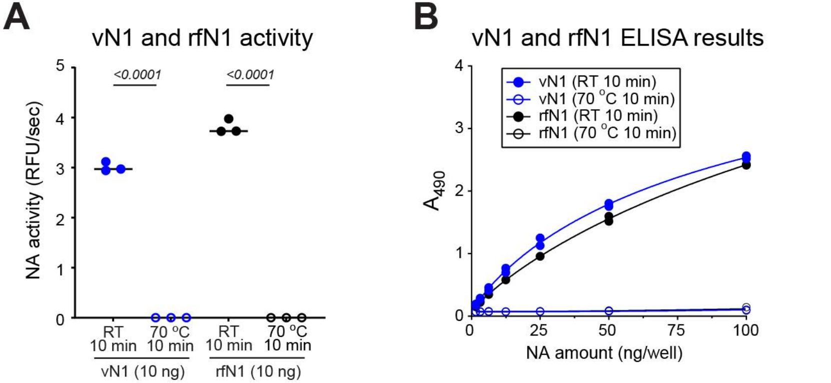

Figure S1. *A*, vN1 and rfN1 were incubated at room temperature (RT) or at 70 °C for 10 min and the activity was measured in triplicate using MUNANA. *P* values are from an unpaired student t-test. *B*, Results from an N1 sandwich ELISA run in duplicate with the indicated amounts of vN1 and rfN1 incubated either at RT or 70 °C for 10 min are displayed.

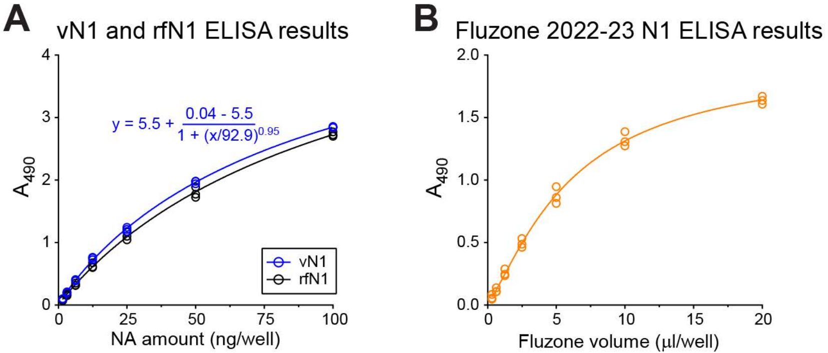

Figure S2. Results from an N1 sandwich ELISA run in triplicate with the indicated amounts of (*A*) vN1 and rfN1 are displayed separately from (*B*) results obtained using the indicated volume of an expired Fluzone lot from the 2022-23 season. All results were obtained at the same time using the same plate and the curve was determined by a 4-PL regression analysis. The 4-PL equation for vN1 (shown in *A*) was used to determine the NA content in the vaccine samples.

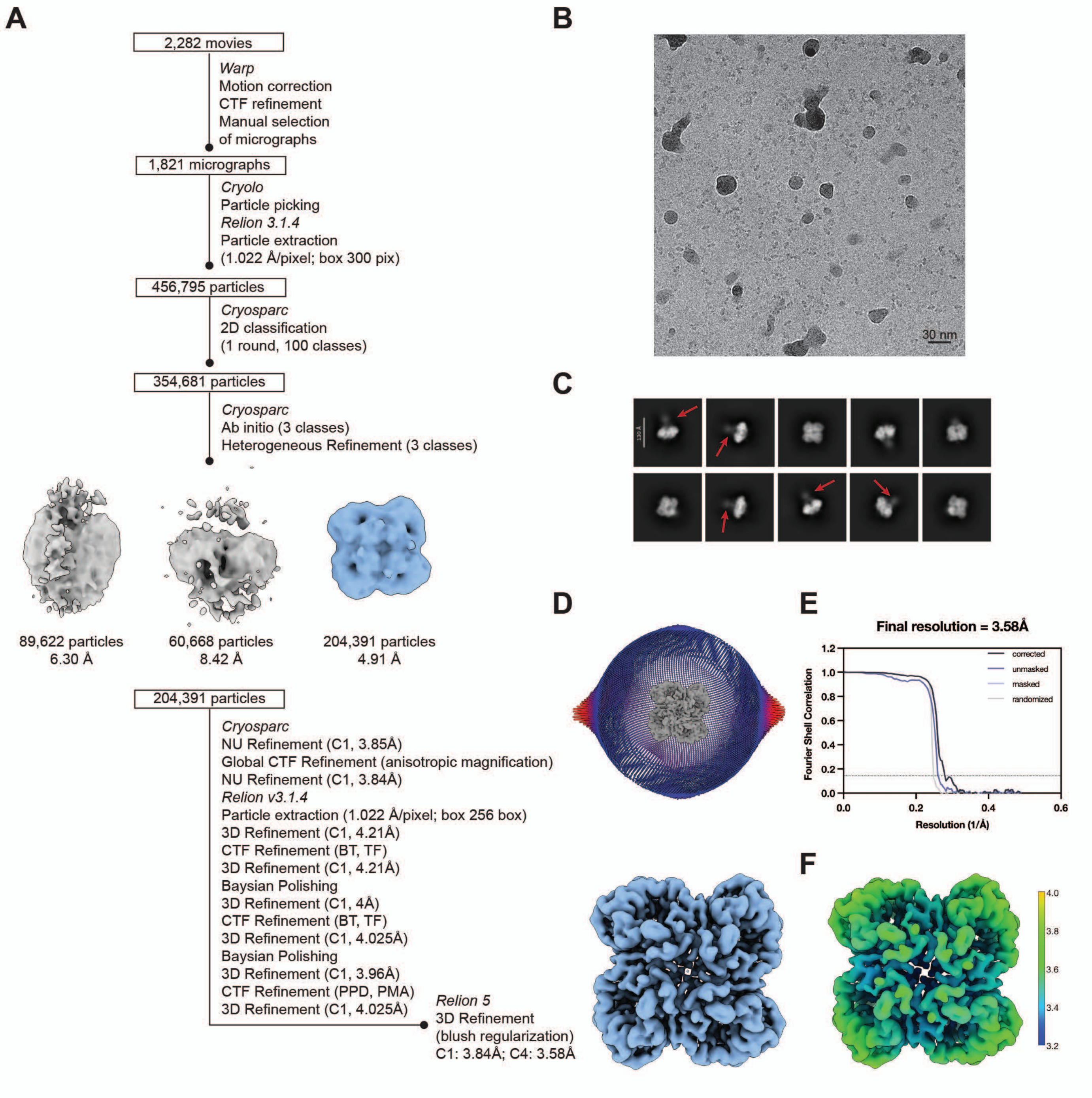

Figure S3. Cryo-EM analysis of viral N1 in HDM detergent. *A,* Image processing workflow with the final C4-symmetrized cryo-EM reconstruction. Abbreviations: BT = beam tilt, TF = trefoil, PPD = per-particle defocus, PMA = per-micrograph astigmatism. *B*, Representative cryo-EM micrograph showing the vN1 particle distribution. *C*, Representative 2D class averages with the vN1 stalk region indicated by red arrows. *D*, Angular distribution of particles contributing to the vN1 reconstruction in C1 symmetry. *E*, Fourier shell correlation plot (0.143 criteria) of C4-symmetrized vN1 map. *F*, Local resolution of C4-symmetrized vN1 map.

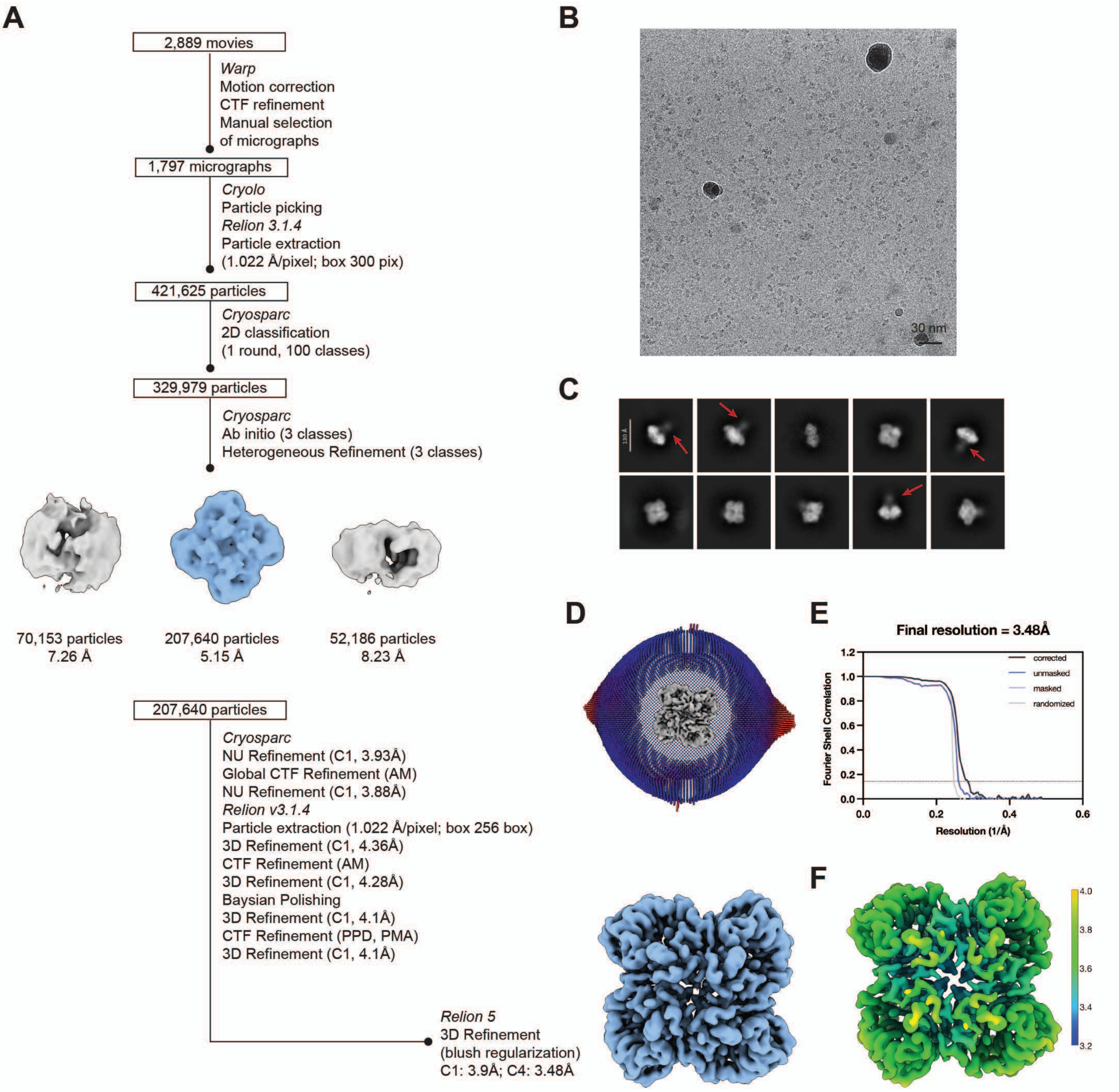

Figure S4. Cryo-EM analysis of viral N1 (vN1) in complex with the inhibitor Zana in HDM detergent (vN1-Zana). *A*, Image processing workflow with the final C4-symmetrized cryo-EM reconstruction. Abbreviations: BT = beam tilt, TF = trefoil, PPD = per-particle defocus, PMA = per-micrograph astigmatism. *B*, Representative cryo-EM micrograph showing the vN1-Zana particle distribution. *C*, Representative 2D class averages with vN1 stalk region indicated by red arrows. *D*, Angular distribution of particles contributing to the vN1-Zana reconstruction in C1 symmetry. *E*, Fourier shell correlation plot (0.143 criteria) of C4-symmetrized vN1-Zana map. *F*, Local resolution of C4-symmetrized vN1-Zana map.

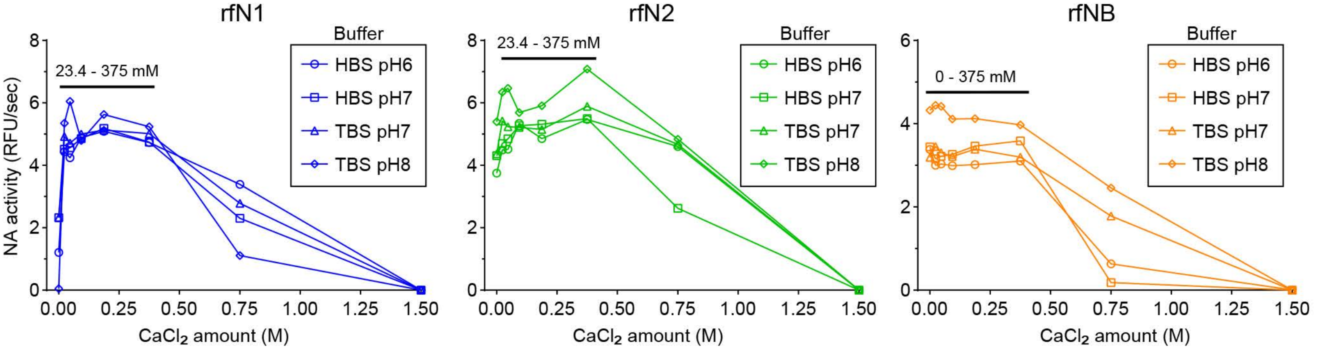

Figure S5. Buffer screeens for $CaCl_2$ resistance. Fixed amounts of rfN1 (left panel), rfN2 (middle panel) and rfNB (right panel) were incubated at room temperature for 22 h in the indicated buffers with increasing $CaCl_2$ concentrations prior to measuring the NA activity with MUNANA. Analysis was performed with HEPES buffered saline (HBS) pH 6 and 7, and Tris buffered saline (TBS) pH 7 and 8. Lines inidcate $CaCl_2$ ranges where activity appeared stable after the 22 h incubation.

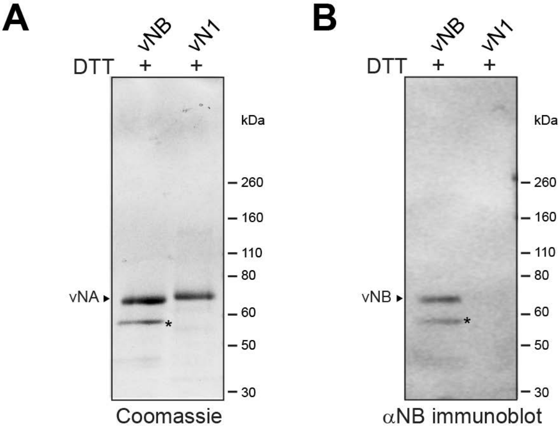

Figure S6. *A*, Coomassie stained SDS-PAGE gel (4-12%) of the isolated vNB and vN1 reduced with DTT prior to resolution. *B*, Immunoblots of the same DTT treated vNB and vN1 samples resolved on the same SDS-PAGE gel and probed with antisera raised against heat denatured recombinant vNB. Asterisks indicate the lower vNB molecular weight band.

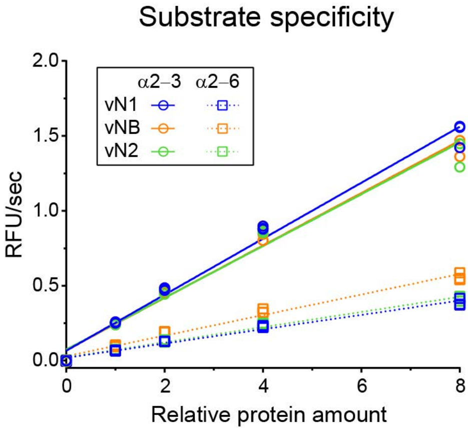

Figure S7. Relative sialyl linkage-specific cleavage activity of the vNAs for α2–3- and α2–6-linked sialosides were measured with an enzyme coupled assay. Protein amounts were standardized based on α2–3-linked sialic acid activity prior to diluting. Data from three independent runs are shown.
