# Supplementary material for: INAAC: An affinity chromatography strategy enabling characterization and quantification of influenza neuraminidase antigens in vaccines
Source: J Biol Chem. 2026 May 12;302(7):113138. doi: 10.1016/j.jbc.2026.113138 (PMC13264171; doi:10.1016/j.jbc.2026.113138)
Supplement: Supplementary Movie Legend and Methods [file mmc3.docx]

**SUPPLEMENTARY MOVIE AND METHODS**

**INAAC: An affinity chromatography strategy enabling characterization and quantification of influenza neuraminidase antigens in vaccines**

Hyeog Kang^1^, Anna Borowska^2^, Tapan Kanai^1^, Jin Gao^1^, Hai Yu^3^, Xi Chen^3^, Jason Gorman^1^, Dirk-Jan Slotboom^2^ and Robert Daniels^1*^

**Supplementary Movie S1. Visualization of vN1 Stalk Motion, related to Figure 3.** Movie illustrating the motion of the vN1 stalk relative to the tetrameric head domain. The movie was generated from a series of 3D maps produced by 3D Variability Analysis, which is shown here at a relatively fixed position. The pronounced flexibility is attributed to a combination of a distinct hinge region connecting the stalk to the head, conformational plasticity within the stalk, and the disordered nature of the predicted 20 *N-*linked glycans (5 per stalk).

**Methods for 3D Variability Analysis and Visualization of Stalk Motion**

**Cryo-EM grid preparation and data collection.** For grid preparation of the dataset used for 3D variability, 2.3 µl of vN1 protein in N1 HDM wash buffer at 1.7 mg/ml was applied to a glow-discharged Quantifoil R 1.2/1.3 300-mesh copper-carbon grid. Vitrification was performed using a Leica EM GP plunge freezer. Sample was incubated on the grid at 95% relative humidity for 30 sec at room temperature, blotted for 3 seconds, and plunged into liquid ethane. Vitrified grids were stored in liquid nitrogen until imaging. Grids were imaged on a Thermo Scientific Tundra Cryo-Transmission Electron Microscope at an accelerating voltage of 100-keV. Micrograph movies were acquired in automated mode using Thermo Scientific EPU software. Images were recorded on a Ceta-F camera at a nominal magnification of 140,000x, corresponding to a calibrated pixel size of 0.95 Å/pixel at the specimen level. Data were collected utilizing a wide defocus range of -0.5 to -2.3 µm. Each movie was recorded with a total electron dose of approximately 47 e⁻/Å² fractionated across 41 frames per movie stack.

**3D Variability Analysis and Visualization of Stalk Motion**. All single-particle image processing for the 3D variability analysis was carried out using cryoSPARC v4.7 (Structura Biotechnology Inc.). Raw movie stacks were imported and subjected to patch motion correction to account for beam-induced motion, followed by contrast transfer function (CTF) estimation using patch CTF estimation. Micrographs with poor CTF fits or excessive motion were discarded. An initial set of particles was picked using Blob Picker, extracted, and subjected to reference-free 2D classification to identify structural classes and filter out junk particles and artifacts. Good 2D classes were selected to generate five *ab initio* 3D models. The selected particles were then subjected to heterogeneous refinement to further classify the data in 3D and remove unstructured particles. The most populated and best-resolved class was refined utilizing homogenous refinement. Conformational dynamics of the vN1 stalk was determined by a 3D Variability Analysis (3DVA) in cryoSPARC v4.7.1 (1) using 189,435 particles from a C1 refinement. A wide mask of the entire vN1 tetrameric complex was used to ensure all potential motion was captured. The 3DVA job was run to solve three orthogonal principal components of variability. To focus the analysis on large-scale domain motions and prevent the inclusion of high-frequency noise, the filter resolution was set to 4.0 Å with 20 maps generated for each component. The output components were inspected using Chimera v1.17 (2) and the analysis revealed that component 1 corresponded to a significant hinge-like motion of the stalk domain relative to a more static head domain. A series of 20 maps representing discrete states on a continuous conformational trajectory was generated and recorded as a movie in UCSF Chimera. The 20 maps were loaded as a volume series and displayed at a consistent contour level that best represents the head and stalk region density throughout the motion. We note that the movie displays the head in a static reference and the stalk moving, while *in vivo* the stalk would be anchored to the membrane leaving the head as the primary component in motion.

**REFERENCES**

1. Punjani, A., Rubinstein, J. L., Fleet, D. J., andBrubaker, M. A. (2017) cryoSPARC: algorithms for rapid unsupervised cryo-EM structure determination Nat Methods **14**, 290-296 10.1038/nmeth.4169

2. Pettersen, E. F., Goddard, T. D., Huang, C. C., Couch, G. S., Greenblatt, D. M., Meng, E. C. *et al.* (2004) UCSF Chimera--a visualization system for exploratory research and analysis J Comput Chem **25**, 1605-1612 10.1002/jcc.20084
