# Supplementary figures and images for: INAAC: An affinity chromatography strategy enabling characterization and quantification of influenza neuraminidase antigens in vaccines
Source: J Biol Chem. 2026 May 12;302(7):113138. doi: 10.1016/j.jbc.2026.113138 (PMC13264171; doi:10.1016/j.jbc.2026.113138)

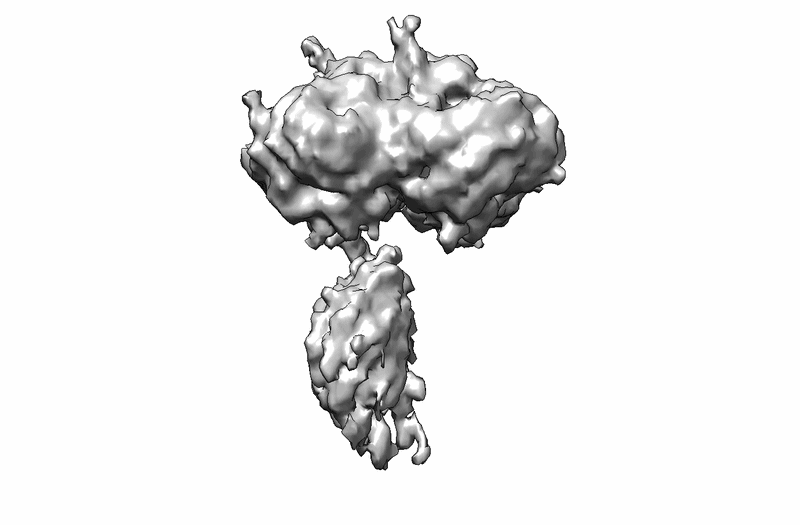

Supplement: Movie S1 [file mmc4.gif]
